# Targeting the GPR183/EBI2‒oxysterol axis in tuberculosis: Immunometabolic regulation and prospects for host‐directed therapy

**DOI:** 10.1002/ctm2.70755

**Published:** 2026-09-11

**Authors:** Junfei Wang, Yingnan Zhou, Xunming Ji, Haiping Zhao, Lingjun Zhan

**Affiliations:** ^1^ Institute of Laboratory Animal Science, Beijing Key Laboratory for Animal Models of Emerging and Remerging Infectious Diseases, Chinese Academy of Medical Sciences and Comparative Medicine Center, Peking Union Medical College Beijing China; ^2^ National Center of Technology Innovation for Animal Model Beijing China; ^3^ State Key Laboratory of Respiratory Health and Multimorbidity Beijing China; ^4^ Key Laboratory of Pathogen Infection Prevention and Control (Peking Union Medical College) Ministry of Education Beijing China; ^5^ State Key Laboratory of Respiratory Health and Multicomorbidity Beijing China; ^6^ Neuro‐Cardio‐Vascular Diseases Research Laboratory and Beijing Geriatric Medical Research Center Xuanwu Hospital of Capital Medical University Beijing China

**Keywords:** autophagy, GPR183/EBI2, host‐directed therapy, *Mycobacterium tuberculosis*, oxysterol, type I interferon

## Abstract

**Background:**

Tuberculosis (TB), caused predominantly by *Mycobacterium tuberculosis* (*Mtb*), remains a major global health challenge despite the availability of antimicrobial chemotherapy. Drug‐resistant TB, latent infection, immunopathology and metabolic comorbidities such as diabetes continue to undermine treatment efficacy, highlighting the urgent need for host‐directed therapeutic (HDT) strategies that complement antibacterial regimens. G protein‐coupled receptors (GPCRs) are highly tractable drug targets that integrate immune and metabolic signals. Among them, GPR183, also known as Epstein‒Barr virus‐induced gene 2 (EBI2), has emerged as an oxysterol‐sensing receptor with growing relevance to TB pathogenesis.

**Main body:**

GPR183 is activated by oxysterol gradients, particularly 7α,25‐dihydroxycholesterol (7α,25‐OHC), which is generated via the CH25H‒CYP7B1‒HSD3B7 metabolic axis. This pathway regulates diverse immune functions, including immune‐cell positioning, macrophage recruitment, dendritic‐cell and lymphocyte localisation, type I interferon restraint and autophagy induction. In the context of TB, reduced GPR183 expression and impaired oxysterol signalling have been linked to disease severity, diabetes‐associated susceptibility and defective macrophage antimicrobial responses.

**Conclusion:**

Collectively, these observations position the GPR183‒oxysterol axis as a potential immunometabolic checkpoint that coordinates macrophage trafficking with autophagic control of intracellular *Mtb* while simultaneously curbing excessive type I interferon‐driven immunopathology. In this review, we summarise current evidence linking GPR183 biology with TB immunity, discuss available pharmacological modulators and GPCR‐targeted drug‐development challenges, and propose experimental frameworks to evaluate GPR183 as a candidate HDT target for TB.

## Key points

- GPR183/EBI2 links oxysterol metabolism with immune‐cell positioning and macrophage function in tuberculosis.
- The 7α,25‐OHC‐GPR183 axis regulates macrophage trafficking, autophagy and type I interferon responses.
- GPR183 represents a potential immunometabolic target for host‐directed therapy against tuberculosis..
- Pharmacological modulation of GPR183 provides new opportunities for GPCR‐based TB intervention.

## Graphical abstract

1. GPR183/EBI2 links oxysterol metabolism with immune‐cell positioning and macrophage function in tuberculosis.
2. The 7α,25‐OHC‐GPR183 axis regulates macrophage trafficking, autophagy and type I interferon responses.
3. GPR183 represents a potential immunometabolic target for host‐directed therapy against tuberculosis..
4. Pharmacological modulation of GPR183 provides new opportunities for GPCR‐based TB intervention.

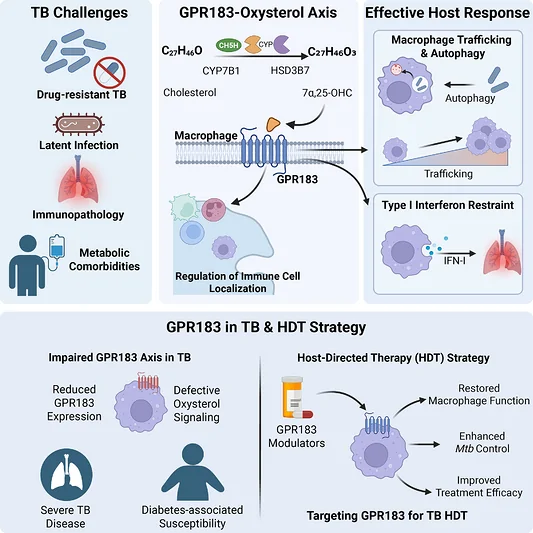

## 1 INTRODUCTION

Tuberculosis (TB) remains one of the most important infectious diseases worldwide. According to the World Health Organisation, approximately 10.7 million people developed TB in 2024 and 1.23 million people died from the disease, including about 150 000 people living with HIV.^1^, ^2^ About one quarter of the global population is estimated to be infected with TB bacteria, most of whom have clinically silent infection but remain at risk of later disease activation. Drug‐resistant TB also continues to be a major public‐health threat, with only about two in five people with drug‐resistant TB accessing treatment in 2024.^2^ These epidemiological features underscore two linked problems: direct antimicrobial therapy is indispensable but insufficient, and host immune status strongly shapes infection outcome.

*Mycobacterium tuberculosis* (*Mtb*) is transmitted mainly by aerosols and primarily establishes infection in the lung. After deposition in the distal airways, bacilli are taken up by alveolar macrophages, interstitial macrophages, monocytes and dendritic cells. Alveolar macrophages are particularly important because they are among the first cellular niches encountered by *Mtb* and represent both a replication site and a platform for innate immune activation. The host response then evolves into a coordinated cellular network involving recruited monocytes, macrophages, neutrophils, dendritic cells, T cells, B cells, innate lymphoid cells (ILCs) and stromal elements. Granuloma formation may restrict bacterial dissemination, but it can also create inflammatory lesions that support persistence, tissue damage, post‐TB lung disease and transmission.^3^ Therefore, TB pathogenesis is not determined solely by bacterial virulence but instead reflects a dynamic balance between mycobacterial survival strategies and host immune–metabolic responses.

Host‐directed therapeutic (HDT) could improve TB outcomes, by modulating host pathways that influence bacterial control, inflammation, tissue repair or treatment tolerance. Unlike antibiotics, HDT does not directly target bacteria and therefore may impose less selective pressure for classical antimicrobial resistance. Several HDT strategies have undergone preclinical experiments, including agents that modulate inflammation, autophagy, eicosanoid balance, matrix remodelling and metabolic dysfunction.^3^, ^4^, ^5^ However, the clinical trials have been challenging because host pathways often have context‐dependent effects. An effective HDT target should meet three key criteria: relevance to *Mtb* infection biology, pharmacological tractability and a well‐defined therapeutic window.

G protein‐coupled receptors (GPCRs) are generally considered more druggable than many other immune targets, owing to their cell‐surface localisation, well‐established assay platforms, tractable ligand‐binding pockets and extensive medicinal chemistry experience.^6^, ^7^, ^8^, ^9^ GPR183/Epstein‒Barr virus‐induced gene 2 (EBI2) is a class A GPCR that detects endogenous oxysterols and regulates immune‐cell localisation and inflammatory signalling. The GPR183‐regulated pathways of macrophage recruitment, autophagy and type I interferon (IFN) signalling substantially overlap with the processes that govern whether *M. tuberculosis* is contained or progresses to disease. This makes the GPR183/oxysterol axis a biologically plausible and druggable candidate for TB HDT.

The central argument of this review is that GPR183 should not be considered only as a general immune‐cell chemotaxis receptor in TB, but also act as an immunometabolic coordinator that links cholesterol metabolism to macrophage positioning, autophagic bacterial restriction and control of IFN‐I‐driven pathology. This framework also clarifies why GPR183 biology appears complex across diseases: the same receptor may be protective in one setting by supporting antimicrobial macrophage function, but pathogenic in another by promoting excessive leukocyte recruitment or autoimmune inflammation. A TB‐focused interpretation therefore requires careful attention to cell type, infection stage, tissue compartment and direction of pharmacological modulation.

## 2 BIOLOGICAL FRAMEWORK OF GPR183/EBI2 SIGNALLING

### 2.1 Receptor discovery, expression and immune relevance

GPR183, originally named EBI2, was first identified as an Epstein‒Barr virus (EBV)‐induced transcript in B cells and was reported to be upregulated by more than 200‐fold in EBV‐infected cells, and it belongs to the rhodopsin‐like class A GPCR family.^7^, ^10^ Early studies revealed that GPR183 signals mainly through pertussis toxin‐sensitive Gαi proteins and is expressed in lymphoid tissues, peripheral blood immune cells and the lung, the prominent expressed cells include B cells, T cells, dendritic cells, macrophages and ILCs, consistent with a role in immune‐cell migration and tissue organisation.^7^

Besides, GPR183 converts local oxysterol gradients into directed immune‐cell positioning and intracellular signalling, which distinguishes GPR183 from classical chemokine receptors because its ligands are cholesterol‐derived metabolites rather than protein chemokines. As a result, GPR183 connects sterol metabolism with immune surveillance, inflammation and tissue‐specific immune organisation.

### 2.2 Oxysterol ligands and the CH25H‒CYP7B1‒HSD3B7 metabolic axis

Two independent studies identified oxysterols as endogenous ligands for GPR183, with 7α,25‐dihydroxycholesterol (7α,25‐OHC) showing particularly strong receptor‐activating activity.^11^, ^12^ This pathway is initiated by cholesterol 25‐hydroxylase (CH25H)‐mediated conversion of cholesterol to 25‐hydroxycholesterol (25‐OHC). Subsequent hydroxylation by CYP7B1 yields 7α,25‐OHC, while HSD3B7 oxidises active dihydroxysterols to 3‐oxo derivatives, thereby attenuating the gradient.^13^ This enzymatic system enables tissues to generate highly localised oxysterol fields that guide GPR183‐expressing cells.

In immune tissues, stromal cells and myeloid cells can contribute to oxysterol production and degradation.^13^, ^14^ In macrophages, inflammatory stimuli such as lipopolysaccharide increase expression of oxysterol‐metabolising enzymes and modulate GPR183 expression.^14^ In the infected lung, this is highly relevant because *Mtb* infection reorganises lipid metabolism and creates microenvironments where macrophages, infected cells and stromal elements may shape GPR183 ligand availability. Therefore, changes in CH25H, CYP7B1 and HSD3B7 should be interpreted not as isolated metabolic events, but as determinants of immune‐cell recruitment and cellular activation.

### 2.3 From Gαi to inflammatory and autophagy pathways

GPR183 activation primarily couples to Gαi signalling, which can inhibit adenylate cyclase and alter cAMP‐dependent pathways. Ligand engagement also induces calcium mobilisation, cytoskeletal rearrangement and MAPK activation, including ERK1/2 and p38 in several cellular contexts.^7^, ^14^, ^15^, ^16^ These signals support directional migration, cell positioning and functional activation. In macrophages, GPR183 signalling has also been connected to suppression of IRF7‐related inflammatory networks and regulation of IFN‐I responses.^17^, ^18^, ^19^, ^20^

For TB, two downstream outputs deserve emphasis. First, GPR183 activation has been reported to enhance autophagy in *Mtb*‐infected macrophages, promoting intracellular bacterial restriction.^19^, ^21^ Second, GPR183 can restrain IFN‐I responses, which are frequently associated with severe TB and impaired antimycobacterial immunity when excessive.^19^, ^22^, ^23^ These two outputs provide a mechanistic bridge between oxysterol sensing and TB outcome: GPR183 may simultaneously support antimicrobial cell‐autonomous defense and prevent maladaptive inflammatory amplification.

### 2.4 Structural and pharmacological features relevant to drug development

As a class A GPCR, GPR183/EBI2 contains the canonical seven‐transmembrane architecture, with extracellular and intracellular loops, an extracellular N‐terminus and an intracellular C‐terminus. Structural understanding of GPR183 has advanced substantially with cryo‐EM structures of both the active 7α,25‐OHC‐bound EBI2–Gi complex and the inactive GSK682753A‐bound receptor.^24^ These structures show that GPR183 contains a deep orthosteric pocket within the transmembrane bundle. In the active state, 7α,25‐OHC is coordinated by key residues including Arg87^^2.60^, Tyr112^^3.33^, Tyr116^^3.37^, Gln162^^4.56^ and Tyr260^^6.51^, which interact with the hydroxyl groups of the oxysterol ligand and contribute to Gi activation.^24^ Earlier mutagenesis studies also identified residues within the transmembrane core, including Arg^^II:20^ (Arg87) and Phe^^VI:13^ (Phe257), as regulators of constitutive activity and receptor activation.^25^

A notable feature of GPR183 is that ligand recognition is influenced not only by the final binding pose but also by ligand entry routes. Molecular dynamics and mutagenesis studies suggest that 7α,25‐OHC preferentially enters the receptor through a lateral membrane‐facing channel between TM4 and TM5, whereas less lipophilic synthetic ligands may also use an extracellular polar water channel.^26^ This dual‐entry model helps explain how GPR183 recognises both endogenous oxysterols and structurally diverse synthetic ligands.

**FIGURE 1:**
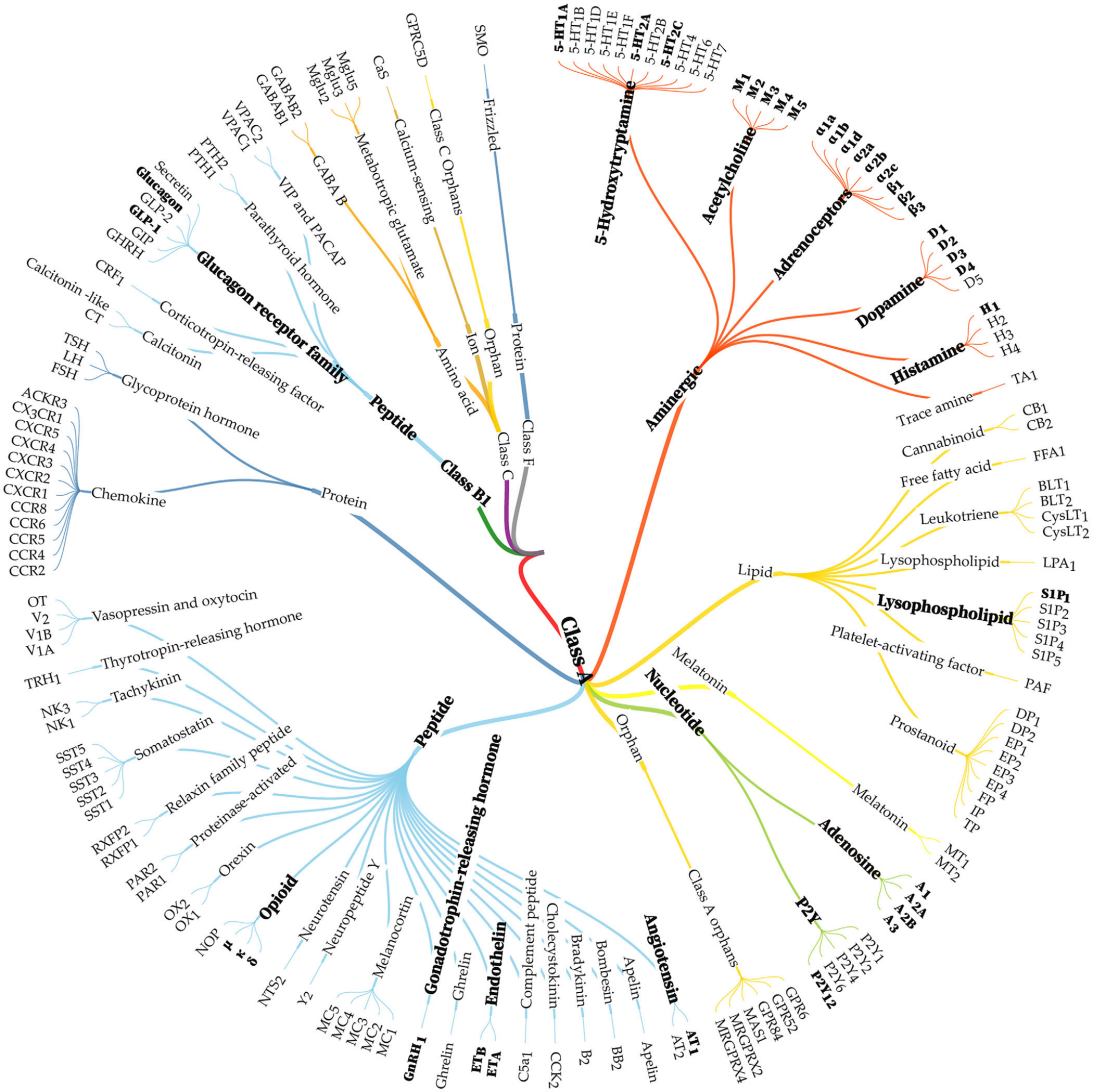
Diversity of G protein‐coupled receptor (GPCR) families and the pharmacological context of GPR183/Epstein‒Barr virus‐induced gene 2 (EBI2). GPCRs comprise a large and functionally diverse receptor superfamily that detects peptides, amines, lipids, nucleotides and other extracellular signals. GPR183/EBI2 belongs to the class A/rhodopsin‐like GPCR group and acts as an oxysterol‐sensing receptor. The diversity of GPCR ligand classes and signalling mechanisms provides a strong pharmacological rationale for targeting GPR183, while also emphasising key development challenges, including tissue specificity, ligand bias, downstream signalling selectivity and long‐term safety. The drug target classification tree was constructed based on the GPCRdb database, with currently priorized targets highlighted in bold.

Pharmacologically, GPR183 can be modulated by agonists, antagonists and inverse agonists. 7α,25‐OHC is the principal endogenous agonist, while GSK682753A, NIBR189 and related compounds are commonly used antagonists or inverse agonists. Interestingly, agonists have also been identified from an antagonist‐derived scaffold, suggesting that subtle structural changes can switch ligand efficacy.^27^ More recent structure‐based screening identified compound 78 as an inverse agonist that inhibits constitutive and agonist‐induced Gi signalling, β‐arrestin2 recruitment and immune‐cell migration; Tyr260^^6.51^ was proposed as a key activation switch involved in efficacy and signalling bias.^28^ These findings support structure‐guided development of GPR183 modulators for TB, but future studies should test whether candidate ligands can selectively enhance macrophage autophagy and bacterial control without promoting excessive inflammatory recruitment (Figure 1).

## 3 KNOWN IMMUNOLOGICAL FUNCTIONS OF GPR183 AND THEIR POTENTIAL IMPLICATIONS IN TB

### 3.1 Macrophages: the central TB‐relevant cell type

Macrophages are the most important cell type for linking GPR183 biology to TB.^19^ *Mtb* enters the lung via the airways, where it is first encountered by alveolar macrophages.^29^, ^30^ These cells can kill bacilli through phagosome maturation, lysosomal fusion, reactive nitrogen and oxygen species, antimicrobial peptides and autophagy.^31^ However, *Mtb* has evolved mechanisms to persist inside macrophages by blocking phagosome‒lysosome fusion, manipulating host lipid metabolism and altering innate signalling.^32^ Thus, the same macrophage can become either an antimicrobial effector cell or a permissive bacterial niche.^33^

The GPR183/oxysterol axis influences macrophage biology at several aspects. It can direct macrophage migration towards oxysterol‐rich inflammatory sites, regulate calcium and MAPK signalling, and modulate inflammatory cytokine production.^14^, ^34^ During *Mtb* infection, impaired GPR183/oxysterol signalling has been associated with delayed macrophage recruitment to the lung and worse disease under dysglycaemic conditions.^35^, ^36^ This is particularly important for TB‒diabetes comorbidity, where altered glucose and lipid metabolism may blunt oxysterol generation and compromise macrophage localisation and function.

The missing connection in many descriptions of GPR183 is the link between migration and macrophage cell‐autonomous defense. Recruitment alone is not sufficient if incoming macrophages are functionally impaired. Conversely, autophagy activation is insufficient if macrophages fail to reach infected foci. GPR183 may be valuable in TB because it potentially integrates both processes: it promotes macrophage positioning through oxysterol gradients and enhances autophagy‐dependent bacterial restriction within infected macrophages.^19^, ^21^ Therefore, future TB studies should assess migration, bacterial burden and autophagic flux together rather than treating them as separate endpoints.

### 3.2 B cells, humoural immunity and lymphoid organisation

GPR183 was initially characterised in B‐cell biology, where it regulates the migration of activated B cells within follicles and supports T‐dependent antibody responses.^13^, ^37^, ^38^ This function is relevant to TB because adaptive immunity in chronic lung infection depends not only on T‐cell activation but also on the structure of local immune niches. Although antibodies are not traditionally viewed as the dominant protective mechanism against intracellular *Mtb*, B cells can modulate TB immunity through antigen presentation, cytokine production, regulation of granuloma architecture and support of ectopic lymphoid structures. These B‐cell‐containing lymphoid aggregates may influence local T‐cell responses and macrophage activation within infected lung tissue.

**FIGURE 2:**
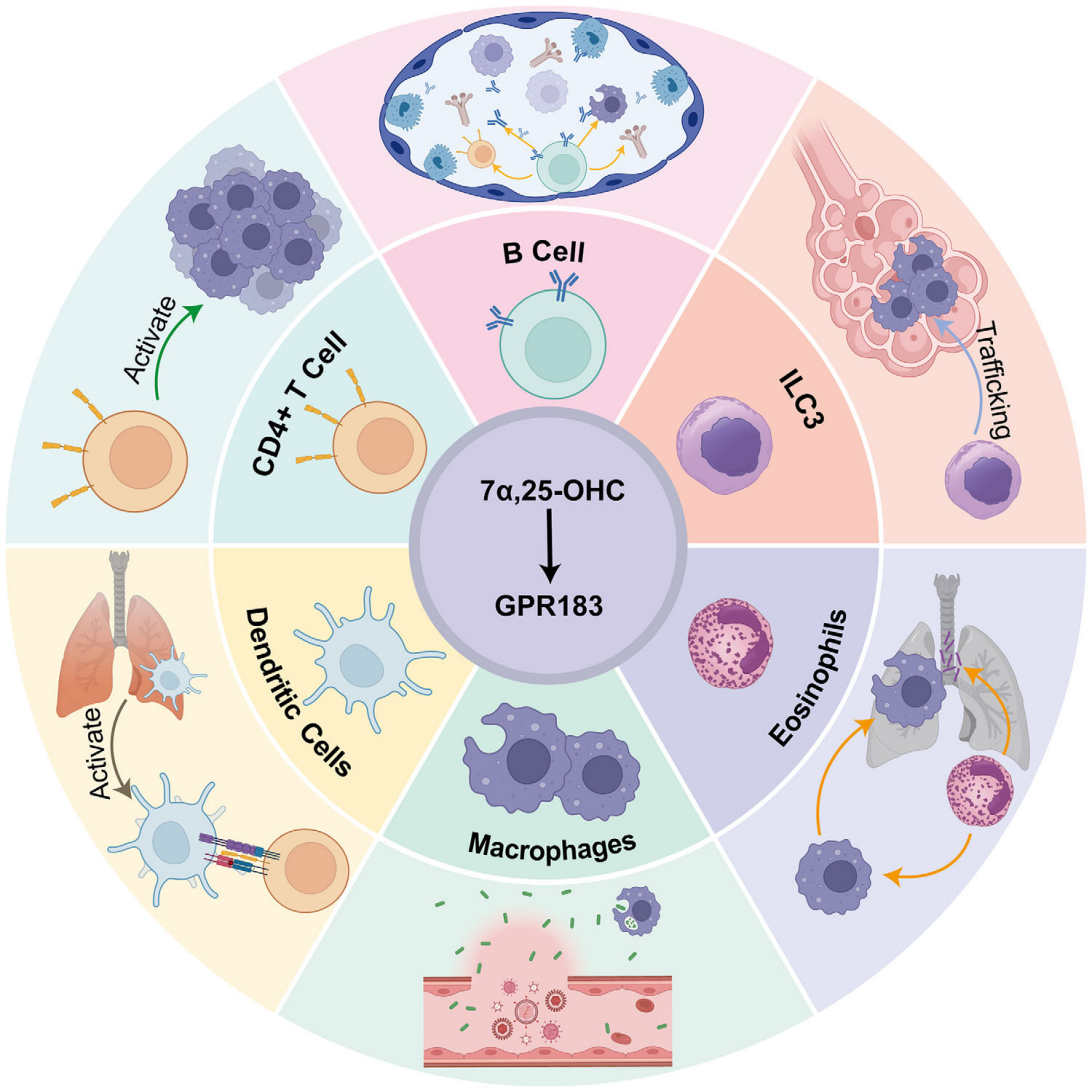
GPR183‐mediated immune‐cell positioning and adaptive immune regulation. GPR183/Epstein‒Barr virus‐induced gene 2 (EBI2) translates local 7α,25‐dihydroxycholesterol (7α,25‐OHC) gradients into spatial organisation of immune cells. In lymphoid tissues, GPR183 contributes to dendritic‐cell positioning, CD4^+^ T‐cell localisation and B‐cell follicular movement, thereby supporting antigen encounter, T‐cell priming and T‐dependent antibody responses. These functions are relevant to TB because effective control of *Mtb* requires coordinated innate and adaptive immunity, including macrophage activation by T‐cell‐derived signals and the organisation of local immune niches in infected lung tissue

Antibody‐mediated immunity in TB is therefore increasingly considered a modulatory component of host defense rather than a strictly sterilising mechanism. Antibodies may affect *Mtb* infection through Fc receptor‐mediated phagocytosis, opsonisation, complement activation, antibody‐dependent cellular functions and regulation of inflammatory cell recruitment. In this context, GPR183‐dependent B‐cell positioning may influence TB immunity through two complementary mechanisms: by supporting T‐dependent antibody responses and by organising local lymphoid microenvironments in which antibody‐dependent, T‐cell‐dependent and macrophage‐mediated immune processes converge. Thus, the relevance of GPR183 to B‐cell biology in TB is likely to extend beyond antibody production alone and may involve broader control of adaptive immune architecture in infected lung tissue (Figure 2).

### 3.3 CD4^+^ T‐cell positioning and Th1 immunity

**FIGURE 3:**
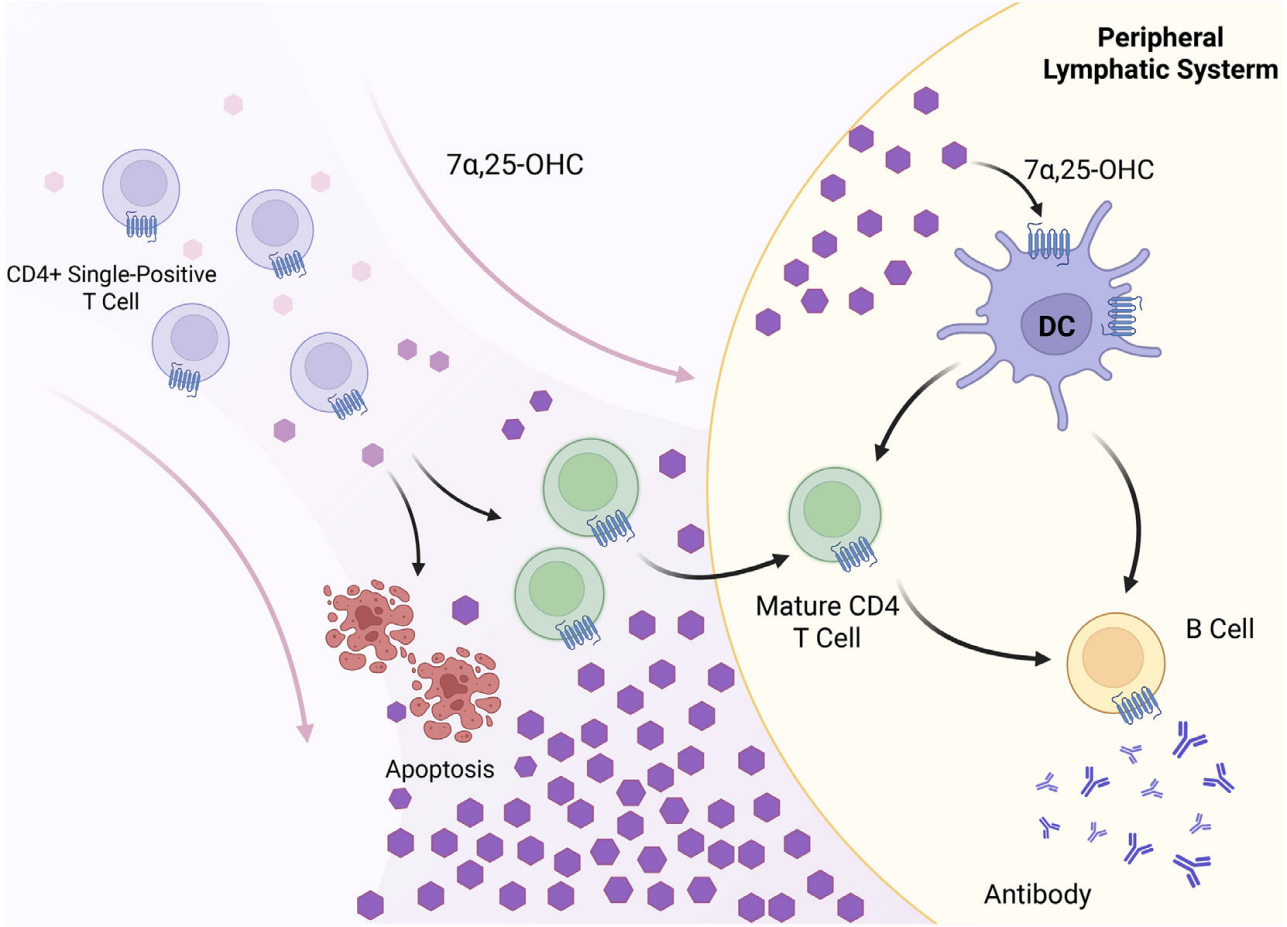
GPR183 mediates thymic negative selection and immune cell coordination in peripheral lymphoid tissues via 7α,25‐OHC signaling. In the thymus, 7α,25‐dihydroxycholesterol (7α,25‐OHC) guides the migration of GPR183‐expressing CD4^+^ single‐positive thymocytes toward the medulla, facilitating their encounter with self‐antigens and promoting apoptosis during negative selectionkey for central tolerance. In the peripheral lymphatic system, 7α,25‐OHC also directs the localization of GPR183^+^ dendritic cells (DCs) to splenic marginal zone bridging channels. These DCs interact with B cells to maintain lymphoid homeostasis and stimulate T cell‐dependent antibody responses, highlighting a dual role of GPR183 in both central immune tolerance and adaptive immunity.

Protective immunity against *Mtb* depends strongly on CD4^+^ T cells, especially IFN‐γ‐producing Th1 cells that activate infected macrophages. GPR183 guides CD4^+^ T‐cell positioning within lymphoid tissues and affects the ability of T cells to encounter antigen‐presenting cells.^39^ This spatial function may influence TB vaccine responses and infection‐induced immunity. The role of CD8^+^ T cells is also relevant in TB, particularly through cytotoxicity and cytokine production, but CD4^+^ T‐cell responses are prioritised because they are more consistently required for macrophage activation and control of intracellular mycobacteria. A balanced discussion should therefore include CD8^+^ T cells while explaining why CD4^+^ T cells remain central to the GPR183‐TB framework (Figure 3).

### 3.4 Dendritic cells and antigen presentation

Dendritic cells contribute to TB immunity by transporting antigen from the lung to draining lymph nodes and initiating T‐cell responses. GPR183 regulates dendritic‐cell positioning in lymphoid tissues and supports their localisation to microanatomical zones needed for efficient antigen capture and T‐cell interaction.^40^, ^41^ In TB, delayed or inefficient antigen presentation can postpone protective T‐cell responses, allowing early bacterial expansion. Although direct evidence for a TB‐specific GPR183 function in dendritic cells remains limited, its established role in dendritic‐cell positioning provides a plausible route through which the oxysterol axis may influence adaptive immune priming.

### 3.5 Eosinophils, ILC3 and other immune cells

GPR183 also regulates eosinophil recruitment to the lung during early *Mtb* infection and allergic inflammation.^42^, ^43^ The consequences of eosinophil recruitment in TB are likely stage‐dependent and require further study. Group 3 ILC3 express GPR183 and migrate toward 7α,25‐OHC, contributing to lymphoid tissue formation and mucosal defense.^44^ Because ILC3 can support early control of *Mtb* by promoting alveolar macrophage accumulation,^44^, ^45^ GPR183 may also influence TB indirectly through ILC3‒macrophage crosstalk. Although osteoclast precursors and γδ T cells are not central to the current TB‐focused framework, their reported dependence on GPR183 further indicates that this receptor may participate in broader tissue remodelling and unconventional immune‐cell regulation (Figure 2).^46^, ^47^

## 4 GPR183 IN TB PATHOGENESIS: CLINICAL AND EXPERIMENTAL EVIDENCE

### 4.1 Reduced GPR183 expression in patients with TB

Clinical observations indicate that reduced GPR183 expression is associated with TB disease severity. GPR183 expression is lower in the blood of TB patients and the expression in TB patients with type 2 diabetes mellitus is lower than that in TB patients without diabetes.^19^, ^35^, ^48^ Lower expression also correlates with more severe radiographic disease in some cohorts.^19^ These findings do not establish causality but support the notion that GPR183 is part of a host‒response module associated with disease progression.

It must be noted that the interpretation of blood GPR183 expression requires caution. Whole‐blood expression reflects both per‐cell transcriptional changes and shifts in immune‐cell composition. Therefore, future clinical studies should combine bulk transcriptomics with cell‐type‐resolved approaches such as flow cytometry, single‐cell RNA sequencing and spatial profiling of lung lesions. It will also be important to distinguish whether decreased blood GPR183 is a marker of disease severity, a contributor to defective immunity, or a consequence of altered immune‐cell distribution during active TB.

### 4.2 TB‒diabetes comorbidity and metabolic impairment of the oxysterol axis

Diabetes is a major risk factor for TB disease and poor treatment outcomes.^2^ The GPR183/oxysterol axis is particularly pertinent to TB–diabetes comorbidity, as it integrates glucose and lipid metabolism with macrophage recruitment and effector function. In dysglycaemic mouse models, *Mtb* infection elicits a blunted GPR183/oxysterol response, diminished macrophage recruitment to the lung, and delayed immune containment.^35^, ^36^ These findings suggest a mechanistic link between metabolic impairment and TB susceptibility that extends beyond generalised immune dysfunction.

More broadly, recent studies further highlight macrophage dysfunction and immune‐metabolic dysregulation in TB‒diabetes, as evidenced by impaired human alveolar macrophage function and altered innate inflammatory control.^49^, ^50^ These data strengthen the rationale for evaluating GPR183 in metabolically stratified TB populations. A clinically viable GPR183‐based HDT strategy is unlikely to be universally applicable across all TB patients; rather, it is most likely to confer benefit in patient subgroups characterised by dysglycaemia, impaired macrophage recruitment, or diminished GPR183/oxysterol pathway activity.

### 4.3 Evidence from mouse and macrophage infection models

Experimental models show that GPR183 deficiency can increase susceptibility to *Mtb* infection. GPR183‐deficient mice exhibit higher bacterial burden and altered inflammatory responses in the lung, while *Mtb*‐infected macrophages show reduced GPR183 expression and changes in autophagy‐ and IFN‐related pathways.^19^, ^21^ Pharmacological activation with 7α,25‐OHC suppresses intracellular *Mtb* growth in macrophages by enhancing autophagy, and this effect can be blocked by GPR183 antagonism.^19^ These findings place GPR183 upstream of a macrophage‒autophagy program with direct antimicrobial relevance.

### 4.4 Disease‐specific features of GPR183 as a TB target

**FIGURE 4:**
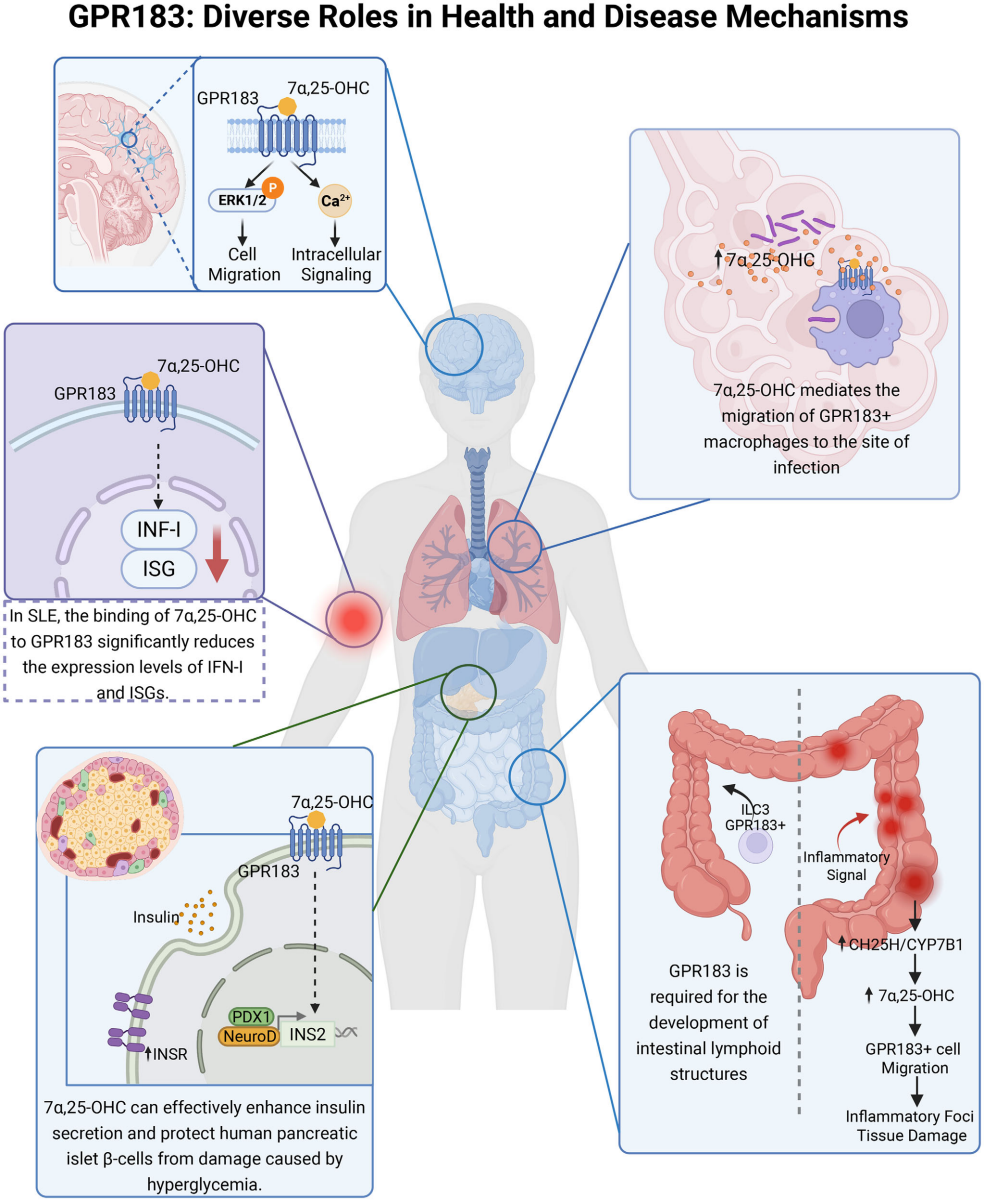
Context‐dependent roles of GPR183/Epstein‒Barr virus‐induced gene 2 (EBI2) across inflammatory, metabolic and infectious diseases. GPR183/EBI2 has been implicated in multiple tissues and disease contexts, including tuberculosis (TB), neuroinflammation, intestinal inflammation, metabolic dysfunction, B‐cell biology, eosinophil recruitment and unconventional T‐cell responses. These examples illustrate that GPR183 signalling can be protective or pathogenic depending on cell type, tissue compartment and disease stage. For TB, the therapeutic value of GPR183 may lie in selectively enhancing macrophage recruitment, autophagy and bacterial control while avoiding excessive inflammatory cell accumulation.

GPR183 has been implicated in autoimmune disease, inflammatory bowel disease, neuroinflammation, pain, metabolic disease and B‐cell malignancy.^16^, ^20^, ^51^, ^52^, ^53^, ^54^, ^55^, ^56^, ^57^ These cross‐disease observations are useful because they show that GPR183 is pharmacologically and biologically important, but they also warn against a simplistic ‘activate’ or ‘inhibit’ strategy. In multiple sclerosis models, GPR183 can promote migration of pathogenic T cells into the central nervous system.^51^, ^52^ In inflammatory bowel disease, GPR183‐dependent immune‐cell positioning may drive colonic inflammation.^55^, ^56^ In systemic lupus erythematosus (SLE), GPR183 appears to restrain IFN‐I and IFN‐stimulated gene expression.^20^, ^53^ In diabetes‐related β‐cell biology, GPR183 activation may enhance insulin secretion and protect against glucotoxicity (Table 1 and Figure 4).^54^

The specificity of GPR183 as a therapeutic target in TB stems from the convergence of three pathophysiologically relevant processes: macrophage trafficking into infected pulmonary tissue, autophagy‐mediated bacterial restriction and suppression of excessive type I IFN responses. In autoimmune or colitis settings, GPR183 inhibition may be desirable when receptor‐driven cellular recruitment amplifies tissue pathology. In TB, however, the optimal therapeutic approach is likely to be more nuanced. Agonism may prove beneficial when macrophage recruitment and autophagy are insufficient, whereas antagonism might be warranted if GPR183‐dependent recruitment promotes deleterious inflammation at particular disease stages or in specific tissue compartments. This context dependence is not an inherent limitation of the target; rather, it constitutes the central translational challenge that must inform experimental design (Figure 4).

**TABLE 1:** Cross‐disease comparison of GPR183 functions and implications for tuberculosis (TB) target development.

Disease context	Main role of GPR183	Therapeutic implication	Relevance to TB
TB	Regulates macrophage recruitment, autophagy and type I IFN responses.	Stage‐ and context‐dependent modulation.	May coordinate bacterial control and limit immunopathology.
Multiple sclerosis/neuroinflammation	Promotes pathogenic immune‐cell trafficking into the CNS(Central nervous system).	GPR183 inhibition may reduce inflammatory recruitment.	Contrasts with TB, where immune‐cell recruitment may be protective.
Inflammatory bowel disease	Supports intestinal immune‐cell positioning and mucosal inflammation.	GPR183 antagonism may suppress excessive inflammation.	Highlights the risk that broad inhibition may impair host defense in TB.
Systemic lupus erythematosus	Restrains type I IFN and ISG activation.	Restoring GPR183 signalling may reduce IFN‐I‐driven pathology.	Similar to TB, where excessive IFN‐I is associated with disease severity.
Diabetes/metabolic dysfunction	Links oxysterol signalling with β‐cell function and metabolic stress.	GPR183 activation may support metabolic homeostasis.	Relevant to TB‒diabetes comorbidity and impaired macrophage responses.
B‐cell malignancy	Alters B‐cell migration, expansion and lymphoid organisation.	Pathway inhibition or normalisation may be beneficial.	Emphasises the need to monitor B‐cell effects during TB‐targeted therapy.

Abbreviations: IFN, interferon; ISG, interferon‐stimulated gene.

## 5 MACROPHAGE AUTOPHAGY AND TYPE I IFN BALANCE

### 5.1 Autophagy and LC3‐associated pathways in *Mtb* infection

Autophagy is a conserved degradation pathway that delivers cytoplasmic material, damaged organelles and intracellular pathogens to lysosomes. In macrophages infected with *Mtb*, autophagy can promote bacterial restriction by enhancing phagosome maturation, lysosomal fusion, antimicrobial peptide delivery and xenophagic targeting of damaged pathogen‐containing compartments.^5^, ^58^, ^59^, ^60^, ^61^, ^62^, ^63^, ^64^ In addition to canonical autophagy, LC3‐associated phagocytosis (LAP) and other non‐canonical LC3 recruitment pathways can modify phagosomes and contribute to antimicrobial defense.^65^, ^66^

*Mtb* actively interferes with these pathways. Virulence systems such as ESX‐1 can promote cytosolic access of bacterial DNA, alter phagosomal integrity and influence both autophagy and inflammatory signalling.^62^, ^67^, ^68^ The outcome is therefore a balance between host degradative defense and bacterial evasion. HDT strategies that restore effective autophagic flux are attractive because they may improve intracellular bacterial clearance without directly targeting bacterial replication. However, autophagy markers must be interpreted carefully. Increased LC3 puncta or LC3‐II can indicate either enhanced autophagosome formation or blocked flux. Rigorous studies should include p62/SQSTM1 turnover, lysosomal colocalisation, tandem mCherry‐GFP‐LC3 reporters, pharmacological flux controls and intracellular CFU(Colony‐forming unit) assays.

### 5.2 GPR183‐mediated autophagy in infected macrophages

GPR183 activation by 7α,25‐OHC enhances autophagy in *Mtb*‐infected macrophages and restricts intracellular bacterial growth.^19^, ^21^ This positions GPR183 as a link between oxysterol metabolism and cell‐autonomous antimicrobial defense. Mechanistically, GPR183 may affect autophagy through Gαi‐dependent signalling, calcium mobilisation, ERK/p38 activation and PI3K‒AKT‒mTOR‐related pathways.^7^, ^14^, ^15^, ^16^, ^69^ In L929 cells, 7α,25‐OHC induces GPR183‐dependent autophagy associated with decreased Akt phosphorylation and increased p53 expression.^69^ Whether the same pathway operates in *Mtb*‐infected primary alveolar macrophages remains unresolved and should be directly tested.

A robust future experiment would combine GPR183 agonism or antagonism with genetic disruption of core autophagy genes, including Atg5, Atg7 or Becn1‐in primary macrophages. If 7α,25‐OHC loses its antimicrobial efficacy upon autophagy blockade, this would provide support for autophagy as a causal downstream mechanism. Conversely, if bacterial restriction persists, alternative mechanisms—such as altered phagocytosis, cytokine production, metabolic reprogramming or regulated cell death—should be systematically evaluated. In parallel, pharmacological or genetic manipulation of downstream signalling nodes (e.g., Akt/mTOR or p53) would help delineate whether GPR183 acts primarily through autophagy or through concurrent pathways. Collectively, such experiments would advance the current correlative association between GPR183 and autophagy towards a more clearly defined causal framework.

### 5.3 Type I IFN in TB: protective signal or pathogenic amplifier?

IFN‐I signalling is essential for antiviral defense, but in TB excessive IFN‐I responses are frequently associated with disease severity and impaired bacterial control.^22^, ^23^ Host recognition of *Mtb* involves multiple pattern‐recognition receptors, including membrane‐associated, endosomal and cytosolic sensing pathways.^70^ Among host pattern‐recognition pathways, cytosolic sensing of *Mtb* DNA through the cGAS‒STING‒TBK1‒IRF3 axis is particularly important for type I IFN induction, leading to IFN‐β production and downstream IFN‐stimulated gene expression.^62^, ^67^, ^71^ While this pathway is part of innate immune detection, sustained IFN‐I can antagonise protective IFN‐γ‐driven macrophage activation, promote inflammatory cell death, alter cytokine balance and create a permissive environment for *Mtb* persistence.

GPR183 has been identified as a negative regulator of IFN‐I‐related pathways. Knockdown or deficiency of GPR183 can increase IRF7 and IFN‐related gene expression, whereas GPR183 activation suppresses IFN‐I production in several immune settings.^17^, ^18^, ^19^, ^20^ In *Mtb* infection, *Gpr183*‐deficient mice show increased lung IRF3 expression and worsened disease. These observations suggest that GPR183 may serve to mitigate deleterious type I IFN amplification during TB. Although these data do not formally establish that GPR183 exerts its protective effect exclusively through IFN‐I suppression, the consistent inverse correlation between GPR183 activity and IFN‐I responses‐coupled with the well‐documented pathogenic role of sustained IFN‐I signalling in TB‐raises the possibility that GPR183 limits disease severity, at least in part, by restraining excessive IFN‐I production. This consideration is particularly pertinent given that host responses conferring benefit early in infection may become pathological when prolonged or excessive.

### 5.4 GPR183 as a rheostat between antimicrobial autophagy and IFN‐I pathology

**FIGURE 5:**
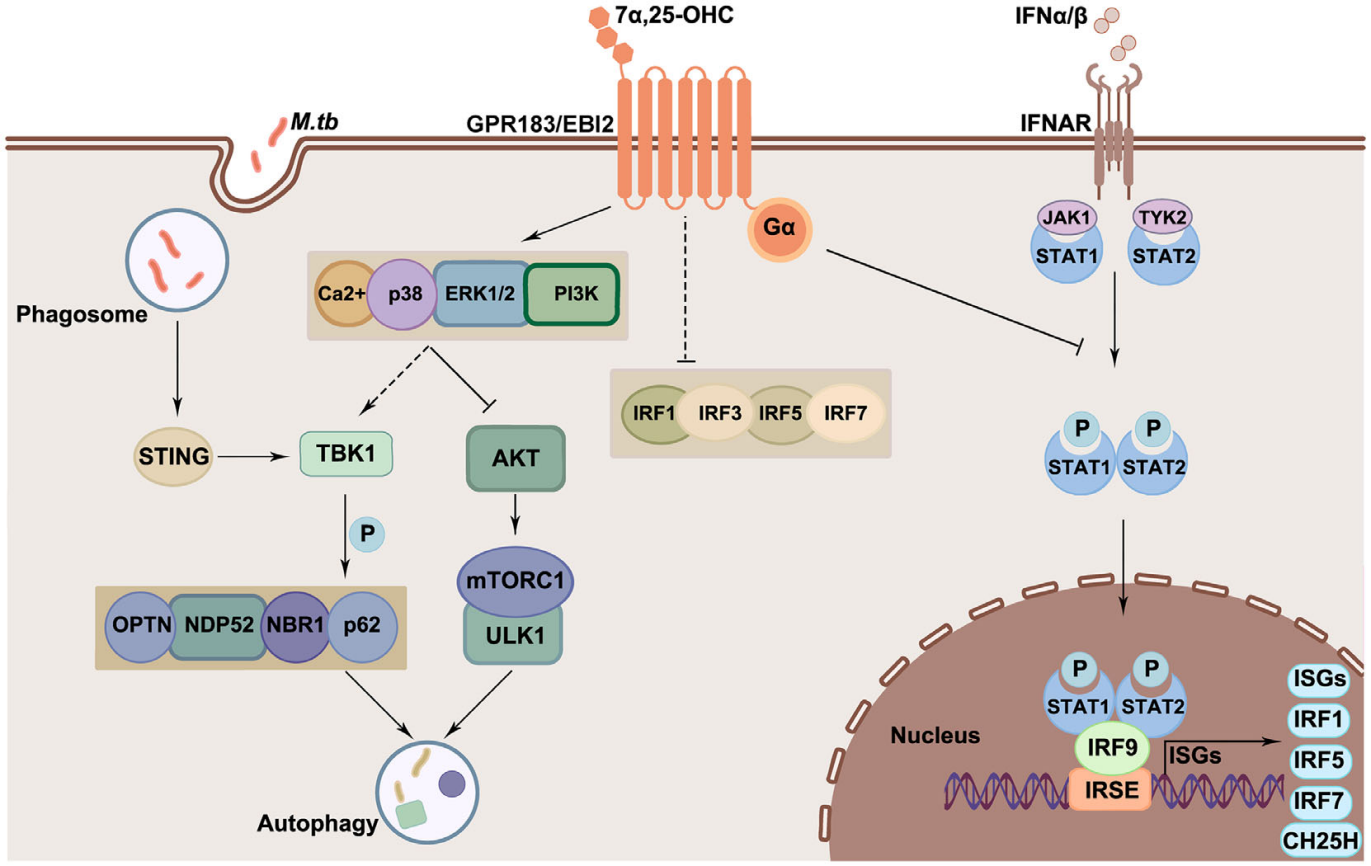
GPR183 signalling and anti‐*Mycobacterium tuberculosis*. As an inhibitory G protein‐coupled receptor, GPR183 signals through classical pathways to activate a series of molecules such as p38, ERK1/2 and PI3K, while inhibiting AKT and enhancing autophagy. Additionally, it may enhance autophagy receptors via TBK1, aiding macrophages in killing *M. tuberculosis*. On the other hand, GPR183 inhibits type I interferon (IFN) signalling through inhibitory G proteins, reducing the expression of interferon‐stimulated genes (ISGs). However, the mechanism by which GPR183 suppresses the expression of various interferon regulatory factors remains unclear. IFNα/β, interferon alpha/beta; IFNAR, interferon alpha and beta receptor; STING, stimulator of interferon genes protein; p38, mitogen‐activated protein kinase 14; ERK1/2, extracellular signal‐regulated protein kinases 1 and 2; PI3K, phosphoinositide 3‐kinase; TBK1, TANK‐binding kinase 1; AKT, protein kinase B; OPTN, optineurin; NDP52, nuclear dot protein 52 kDa; NBR1, neighbour of BRCA1 gene 1; SQSTM1/p62, sequestosome 1; ULK1, UNC51‐like kinase‐1; IRF, interferon regulatory factor; JAK1, janus kinase 1; TYK2, tyrosine kinase 2; STAT1, signal transducer and activator of transcription 1; IRSE, IFN‐stimulated response element; ISGs, interferon‐stimulated genes.

A coherent TB‐centred model is that GPR183 acts as an immunometabolic rheostat. When the CH25H‐CYP7B1 pathway generates appropriate 7α,25‐OHC gradients, GPR183 supports recruitment and positioning of macrophages and other immune cells at infected sites. Within macrophages, GPR183 activation promotes autophagic pathways that help restrict intracellular *Mtb*. At the same time, GPR183 restrains excessive IFN‐I signalling through IRF3/IRF7‐associated networks. The net result may be improved bacterial control with reduced immunopathology (Figure 5).

This model also predicts why dysregulation of the axis is harmful. If GPR183 expression or ligand generation is insufficient, macrophage recruitment may be delayed, autophagic control may be weakened and IFN‐I responses may increase. If the axis is excessively activated in a context dominated by inflammatory recruitment rather than antimicrobial function, tissue damage could be aggravated. Therefore, therapeutic development should not assume that GPR183 agonism is always beneficial or that antagonism is always harmful. Instead, drug testing should stratify by infection stage, metabolic state, lesion type and immune‐cell target.

## 6 PHARMACOLOGICAL MODULATION OF GPR183 AND GPCR DRUG‐DEVELOPMENT CHALLENGES

### 6.1 Overview of approved GPCR‐targeted drugs

**FIGURE 6:**
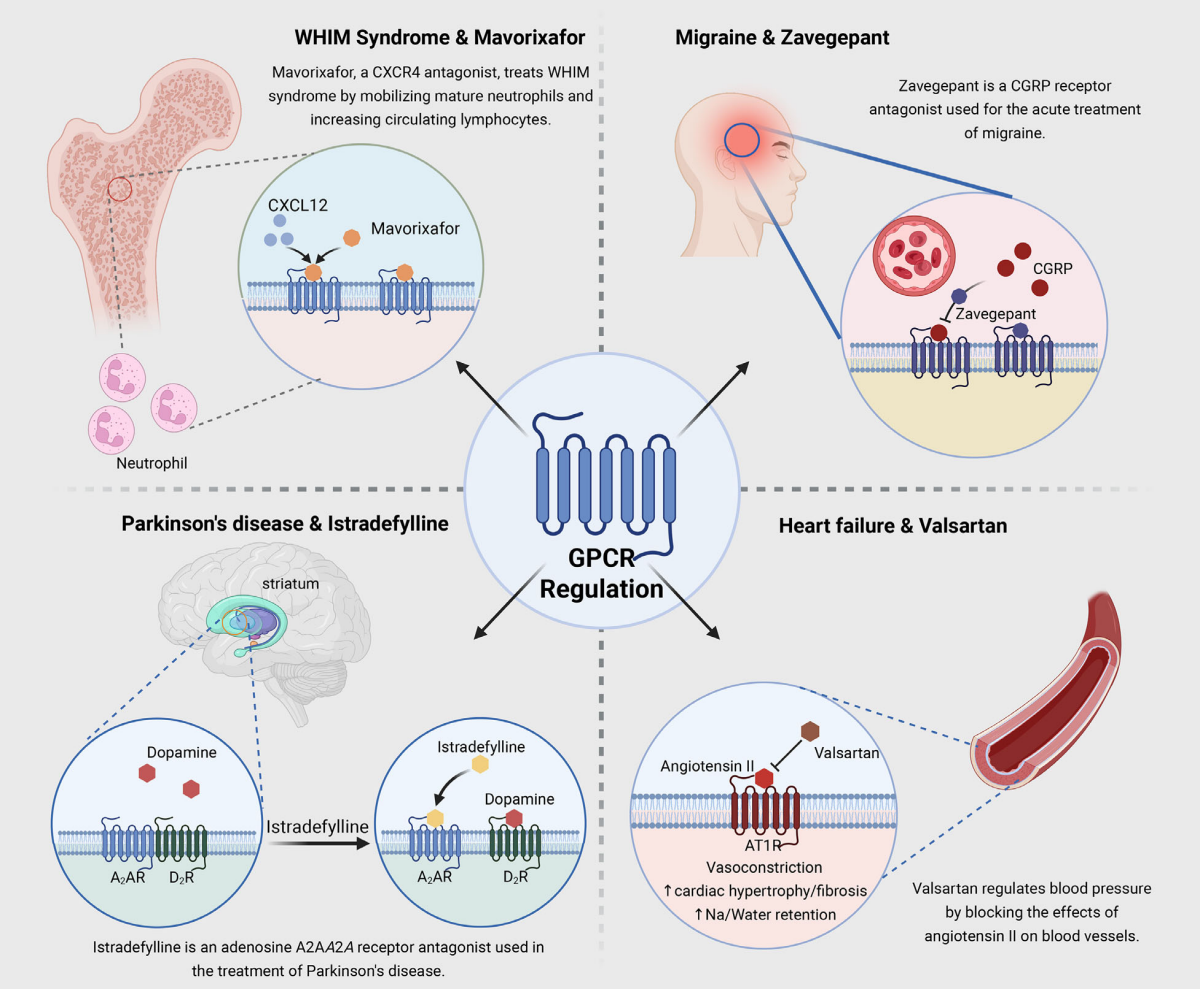
Representative examples of clinically approved G protein‐coupled receptor (GPCR)‐targeted therapies. GPCR‐targeted drugs have been successfully developed for diverse diseases, including WHIM(Wart, hypogammaglobulinemia, infections, and myelokathexis) syndrome, migraine, Parkinson's disease and cardiovascular disease. Examples include mavorixafor targeting CXCR4, zavegepant targeting the CGRP receptor, istradefylline targeting the adenosine A2A receptor and valsartan targeting the angiotensin II type 1 receptor. These cases illustrate the broad clinical tractability of GPCRs and provide a pharmacological rationale for exploring GPR183/EBI2 as a potential target for host‐directed therapy in tuberculosis.

Over the past decade, multiple drugs targeting different GPCR families have been approved for diverse indications, including neurological, cardiovascular, metabolic, inflammatory and endocrine diseases. These approvals demonstrate the successful clinical translation of GPCR modulation across immune, neurological, cardiovascular and metabolic diseases, supporting the broad tractability of GPCRs and the pharmacological rationale for considering GPR183/EBI2 as a potentially druggable target (Figure 6). Representative approved GPCR‐targeted drugs are summarised according to receptor class (Tables S1–S4).

### 6.2 Current GPR183 modulators

The endogenous agonist 7α,25‐OHC remains the key biological tool for activating GPR183. It is useful for mechanistic experiments but has limitations as a therapeutic molecule, including metabolic instability, pleiotropic lipid effects and potential systemic immune consequences. Several antagonists have been reported, including NIBR189, GSK682753, GPR183 antagonist‐1/SAE compounds and newer optimised compounds developed for inflammatory disease contexts.^15^, ^16^, ^19^, ^72^, ^73^ Compound 33, reported in 2024, was designed to improve pharmacokinetic and safety properties and showed promise as an orally active GPR183 antagonist for inflammatory bowel disease models (Table 2).^72^

**TABLE 2:** Representative GPR183 modulators, supporting evidence and their translational relevance to tuberculosis (TB) research.

Modulator	Type	Main reported use or evidence	Relevance to TB development	Key limitation
7α,25‐OHC	Endogenous agonist	Activates GPR183 and promotes immune‐cell migration; enhances autophagy in *Mtb*‐infected macrophages	Tool compound to test whether GPR183 activation improves macrophage autophagy and bacterial control	Limited drug‐like properties; broad oxysterol biology; potential systemic effects
GSK682753	Antagonist	Blocks 7α,25‐OHC‐dependent GPR183 effects in macrophage/TB studies	Useful for confirming receptor dependence in infected macrophages	Limited clinical and in vivo TB validation
NIBR189	Antagonist	Reference GPR183 antagonist used in inflammatory and receptor pharmacology studies	Can test whether blockade worsens or improves TB outcomes depending on stage	May not have ideal pharmacokinetics for chronic TB studies
GPR183 antagonist‐1/SAE series	Antagonist	Reported small‐molecule antagonists with receptor pharmacology activity	Adds chemical diversity for target validation	Needs testing in primary macrophages and *Mtb* infection models
Compound 33	Optimised antagonist	Reported in 2024 as a promising GPR183 antagonist for IBD‐related development	Relevant for druggability and structure‐activity optimisation	Therapeutic direction in TB is uncertain; infection‐stage validation is required

Abbreviations: IBD, inflammatory bowel disease; 7α,25‐OHC, 7α,25‐dihydroxycholesterol.

### 6.3 Challenges in GPCR and GPR183 drug development

Although GPCRs are highly druggable, GPCR drug development faces several challenges. First, GPCR signalling is often pleiotropic. A single GPCR can activate different downstream pathways depending on ligand, cell type and receptor conformation. Second, this pleiotropy makes ligand bias therapeutically attractive but difficult to engineer. For GPR183, an ideal TB‐directed compound would preferentially enhance macrophage autophagy while avoiding excessive inflammatory recruitment. Third, tissue distribution poses additional hurdles: systemic activation or inhibition of GPR183 could affect B‐cell positioning, T‐cell migration, intestinal immunity, neuroinflammation and metabolic function. Fourth, chronic TB therapy may require prolonged treatment windows, making safety and pharmacokinetics profiles key priorities.

These challenges do not preclude GPR183 development; they define the experiments needed. Medicinal chemistry should be integrated with functional assays that distinguish chemotaxis, autophagy, IFN‐I suppression and cytokine production. Receptor pharmacology should include Gαi signalling, β‐Arrestin recruitment, calcium flux, cAMP inhibition, ERK/p38 activation and primary‐cell validation. Structural studies such as cryo‐electron microscopy, homology modelling, AlphaFold‐guided docking, and molecular dynamics simulations could help explain ligand‐binding modes and guide the design of biased or tissue‐selective modulators. Importantly, in vivo pharmacokinetic and pharmacodynamic profiling in relevant TB models will be essential to assess tissue distribution and off‐target effects, particularly in lymphoid organs, the gut, and the central nervous system where GPR183 exerts physiological functions.

## 7 EXPERIMENTAL ROADMAP FOR EVALUATING GPR183 AS A TB HDT TARGET

Future studies should establish the causal role of GPR183 in TB immunity. First, cell‐specific functions need to be defined. Whole‐body *Gpr183* knockout models are informative but cannot distinguish macrophage‐intrinsic effects from changes in dendritic cells, lymphocytes or tissue immune organisation. Conditional and inducible models targeting macrophages, dendritic cells and selected lymphocyte subsets should therefore be combined with low‐dose aerosol *Mtb* infection to determine when and in which cell types GPR183 is protective or pathogenic.

Second, human‐relevant models should be prioritised. Because alveolar macrophages are the first major host cell encountered by *Mtb*, future work should use human alveolar macrophages, alveolar‐like macrophage systems and lung organoid or air‐liquid interface cocultures. These models should also incorporate normoglycemic, hyperglycaemic and diabetic conditions, since metabolic dysfunction may impair the GPR183/oxysterol axis.

Third, mechanistic studies should examine macrophage recruitment, autophagy and type I IFN regulation in the same experimental framework. GPR183 agonists, antagonists or inverse agonists should be tested in *Mtb*‐infected primary macrophages, with parallel assessment of intracellular CFU, autophagic flux, lysosomal delivery, IFNB1/ISG expression and inflammatory cytokines. To establish causality, these pharmacological manipulations should be combined with pathway‐selective interruptions‐such as autophagy inhibition (e.g., ATG5/7 knockdown), STING‒TBK1 blockade, or IFNAR neutralisation‐to determine whether the observed GPR183‐dependent effects are mediated through these downstream mechanisms.

Finally, single‐cell and spatial approaches should be used to define where the GPR183/oxysterol axis operates within infected lung tissue. Pharmacological development should aim not merely to activate or inhibit GPR183, but to identify modulators that enhance macrophage antimicrobial function while avoiding potential off‐target effects on inflammatory cell recruitment. This integrated strategy would clarify whether GPR183 can be advanced as a precision HDT target for TB.

## 8 DISCUSSION

GPR183/EBI2 offers a valuable conceptual entry point for reconsidering TB pathogenesis from an immunometabolic perspective. Rather than functioning merely as a chemotactic receptor, GPR183 integrates cholesterol‐derived oxysterol signals with immune cell positioning, macrophage antimicrobial activity and inflammatory regulation. This is particularly relevant to TB, in which disease outcome is determined not solely by bacterial replication but also by the quality, timing and spatial organisation of host immune responses. Current evidence indicates that reduced GPR183 expression or impaired oxysterol signalling may be associated with defective macrophage recruitment, attenuated autophagic control of intracellular *Mtb*, and excessive type I IFN activity. These observations support the concept that the GPR183/oxysterol axis may function as a host regulatory pathway that coordinates bacterial containment with the limitation of immunopathology.

However, the therapeutic interpretation of GPR183 biology in TB remains complex. In certain inflammatory diseases, GPR183‐driven leukocyte recruitment may exacerbate tissue inflammation, whereas in TB, appropriate macrophage recruitment and activation are likely to be protective. Consequently, GPR183 should not be regarded as a target amenable to simple activation or inhibition; its function is likely contingent upon infection stage, tissue compartment, metabolic status, and the specific immune cell population being targeted. This context dependence is especially critical in TB‒diabetes comorbidity, where altered glucose and lipid metabolism may impair oxysterol generation and macrophage function. In such settings, restoration of GPR183 signalling may prove beneficial, yet excessive or poorly timed activation could, in principle, aggravate inflammatory cell accumulation and pulmonary injury.

Several fundamental questions must be resolved before GPR183 can be advanced as a HDT target. First, it remains to be determined whether reduced GPR183 expression in TB patients represents a causal contributor to disease progression, a biomarker of immune dysregulation, or a consequence of altered immune cell composition. Second, cell‐specific functions require clarification through conditional and inducible models, particularly in macrophages, dendritic cells and lymphocyte subsets. Third, pharmacological studies should extend beyond single‐ligand validation to encompass structurally distinct agonists, antagonists, inverse agonists, and potentially biased modulators. These compounds must be evaluated not only for receptor signalling properties but also for TB‐relevant functional outcomes, including macrophage chemotaxis, autophagic flux, type I IFN regulation, intracellular bacterial burden and pulmonary pathology.

In summary, GPR183 represents a biologically plausible and pharmacologically tractable candidate for host‐directed therapy in TB; however, its therapeutic value will be contingent upon precise disease context. The most promising strategy may not entail broad modulation of GPR183 activity but rather selective enhancement of macrophage‐mediated bacterial containment while avoiding excessive inflammatory recruitment. Future studies will be essential to integrate human alveolar macrophage models, metabolically stratified patient cohorts, spatial profiling of lung lesions, and structure‐guided ligand development. Upon resolution of these challenges, precision targeting of GPR183 becomes increasingly feasible, particularly for TB patients with metabolic disorders or impaired macrophage immune function.

## AUTHOR CONTRIBUTIONS

Junfei Wang and Yingnan Zhou contributed equally to this work as co‐first authors. Junfei Wang was responsible for conceptualisation, literature retrieval and writing the original draft. Yingnan Zhou was responsible for the design and preparation of figures, as well as manuscript review and editing. Haiping Zhao and Lingjun Zhan contributed equally as co‐corresponding authors. Haiping Zhao and Xunming Ji contributed to conceptualisation, validation and critical revision of the manuscript. Lingjun Zhan provided supervision, project administration and funding acquisition. All the authors read and approved the final manuscript.

## CONFLICT OF INTEREST STATEMENT

The authors declare they have no conflicts of interest.

## DATA AVA ILABILITY STATEMENT

Data sharing is not applicable to this article as no datasets were generated or analysed during the current study.

## ETHICS STATEMENT

The authors have nothing to report.

## Supporting information



Supporting Information:

## References

[1] World Health O . Global Tuberculosis Report 2025 . 2025. 978‐92‐4‐011692‐4. 2025/11/12.

[2] World Health O . Tuberculosis. Fact sheets. 2026/03/24 . 2026.

[3] Ofori‐Anyinam B , Nyiro B , Salgame P . Post‐TB lung disease: pathogenesis, host‐directed therapies, and the C3HeB/FeJ model for pre‐clinical advances. Infect Immun. 2026;94(3):e0052324. 41661106 10.1128/iai.00523-24 PMC12974129

[4] Wallis RS , Ginindza S , Beattie T , et al. Adjunctive host‐directed therapies for pulmonary tuberculosis: a prospective, open‐label, phase 2, randomised controlled trial. Lancet Respir Med. 2021;9(8):897‐908. 33740465 10.1016/S2213-2600(20)30448-3 PMC8332197

[5] Kiran D , Podell BK , Chambers M , Basaraba RJ . Host‐directed therapy targeting the *Mycobacterium tuberculosis* granuloma: a review. Semin Immunopathol. 2016;38(2):167‐183. 26510950 10.1007/s00281-015-0537-x PMC4779125

[6] Santos R , Ursu O , Gaulton A , et al. A comprehensive map of molecular drug targets. Nat Rev Drug Discov. 2017;16(1):19‐34. 27910877 10.1038/nrd.2016.230 PMC6314433

[7] Rosenkilde MM , Benned‐Jensen T , Andersen H , et al. Molecular pharmacological phenotyping of EBI2. An orphan seven‐transmembrane receptor with constitutive activity. J Biol Chem. 2006;281(19):13199‐13208. 16540462 10.1074/jbc.M602245200

[8] Munk C , Isberg V , Mordalski S , et al. GPCRdb: the G protein‐coupled receptor database—an introduction. Br J Pharmacol. 2016;173(14):2195‐2207. 27155948 10.1111/bph.13509 PMC4919580

[9] Yuan W , Shi X , Lee LTO . RNA therapeutics in targeting G protein‐coupled receptors: recent advances and challenges. Mol Ther Nucleic Acids. 2024;35(2):102195. 38741614 10.1016/j.omtn.2024.102195 PMC11089380

[10] Birkenbach M , Josefsen K , Yalamanchili R , Lenoir G , Kieff E . Epstein‐Barr virus‐induced genes: first lymphocyte‐specific G protein‐coupled peptide receptors. J Virol. 1993;67(4):2209‐2220. 8383238 10.1128/jvi.67.4.2209-2220.1993 PMC240341

[11] Liu C , Yang XV , Wu J , et al. Oxysterols direct B‐cell migration through EBI2. Nature. 2011;475(7357):519‐523. 21796211 10.1038/nature10226

[12] Hannedouche S , Zhang J , Yi T , et al. Oxysterols direct immune cell migration via EBI2. Nature. 2011;475(7357):524‐527. 21796212 10.1038/nature10280 PMC4297623

[13] Yi T , Wang X , Kelly LM , et al. Oxysterol gradient generation by lymphoid stromal cells guides activated B cell movement during humoral responses. Immunity. 2012;37(3):535‐548. 22999953 10.1016/j.immuni.2012.06.015 PMC3465460

[14] Preuss I , Ludwig MG , Baumgarten B , et al. Transcriptional regulation and functional characterization of the oxysterol/EBI2 system in primary human macrophages. Biochem Biophys Res Commun. 2014;446(3):663‐668. 24480442 10.1016/j.bbrc.2014.01.069

[15] Benned‐Jensen T , Madsen CM , Arfelt KN , et al. Small molecule antagonism of oxysterol‐induced Epstein‒Barr virus induced gene 2 (EBI2) activation. FEBS Open Bio. 2013;3:156‐160. 10.1016/j.fob.2013.02.003 PMC3668520 23772388

[16] Braden K , Giancotti LA , Chen Z , et al. GPR183‒oxysterol axis in spinal cord contributes to neuropathic pain. J Pharmacol Exp Ther. 2020;375(2):367‐375. 32913007 10.1124/jpet.120.000105 PMC7592849

[17] Heinig M , Petretto E , Wallace C , et al. A trans‐acting locus regulates an anti‐viral expression network and type 1 diabetes risk. Nature. 2010;467(7314):460‐464. 20827270 10.1038/nature09386 PMC3657719

[18] Chiang EY , Johnston RJ , Grogan JL , EBI2 is a negative regulator of type I interferons in plasmacytoid and myeloid dendritic cells. PLoS One. 2013;8(12):e83457. 24386204 10.1371/journal.pone.0083457 PMC3873289

[19] Bartlett S , Gemiarto AT , Ngo MD , et al. GPR183 regulates interferons, autophagy, and bacterial growth during *Mycobacterium tuberculosis* infection and is associated with TB disease severity. Front Immunol. 2020;11:601534. 33240287 10.3389/fimmu.2020.601534 PMC7677584

[20] Zhang F , Zhang B , Ding H , et al. The oxysterol receptor EBI2 links innate and adaptive immunity to limit IFN response and systemic lupus erythematosus. Adv Sci (Weinh). 2023;10(27):e2207108. 37469011 10.1002/advs.202207108 PMC10520634

[21] Tang J , Shi Y , Zhan L , Qin C. Downregulation of GPR183 on infection restricts the early infection and intracellular replication of mycobacterium tuberculosis in macrophage. Microb Pathog. 2020;145:104234. 32353576 10.1016/j.micpath.2020.104234

[22] Teles RM , Graeber TG , Krutzik SR , et al. Type I interferon suppresses type II interferon‐triggered human anti‐mycobacterial responses. Science. 2013;339(6126):1448‐1453. 23449998 10.1126/science.1233665 PMC3653587

[23] Donovan ML , Schultz TE , Duke TJ , Blumenthal A. Type I interferons in the pathogenesis of tuberculosis: molecular drivers and immunological consequences. Front Immunol. 2017;8:1633. 29230217 10.3389/fimmu.2017.01633 PMC5711827

[24] Chen H , Huang W , Li X . Structures of oxysterol sensor EBI2/GPR183, a key regulator of the immune response. Structure. 2022;30(7):1016‐1024.e5. 35537452 10.1016/j.str.2022.04.006 PMC9271633

[25] Benned‐Jensen T , Rosenkilde MM. Structural motifs of importance for the constitutive activity of the orphan 7TM receptor EBI2: analysis of receptor activation in the absence of an agonist. Mol Pharmacol. 2008;74(4):1008‐1021. 18628402 10.1124/mol.108.049676

[26] Kjær VMS , Stępniewski TM , Medel‐Lacruz B , et al. Ligand entry pathways control the chemical space recognized by GPR183. Chem Sci. 2023;14(39):10671‐10683. 37829039 10.1039/d2sc05962b PMC10566501

[27] Kjaer VMS , Ieremias L , Daugvilaite V , et al. Discovery of GPR183 agonists based on an antagonist scaffold. ChemMedChem. 2021;16(17):2623‐2627. 34270165 10.1002/cmdc.202100301 PMC8518411

[28] Andersson L , Roggia M , Wangriatisak K , et al. Structure‐based screening and a conformational biosensor identify a GPR183 inverse agonist and an activation switch. Nat Commun. 2026;17(1):7020. 42218170 10.1038/s41467-026-73857-9 PMC13392048

[29] Mai D , Jahn A , Murray T , et al. Exposure to Mycobacterium remodels alveolar macrophages and the early innate response to *Mycobacterium tuberculosis* infection. PLoS Pathog. 2024;20(1):e1011871. 38236787 10.1371/journal.ppat.1011871 PMC10796046

[30] Cohen SB , Gern BH , Delahaye JL , et al. Alveolar macrophages provide an early *Mycobacterium tuberculosis* niche and initiate dissemination. Cell Host Microbe. 2018;24(3):439‐446.e4. 30146391 10.1016/j.chom.2018.08.001 PMC6152889

[31] Maphasa RE , Meyer M , Dube A. The macrophage response to *Mycobacterium tuberculosis* and opportunities for autophagy inducing nanomedicines for tuberculosis therapy. Front Cell Infect Microbiol. 2020;10:618414. 33628745 10.3389/fcimb.2020.618414 PMC7897680

[32] Chai Q , Wang L , Liu CH , Ge B. New insights into the evasion of host innate immunity by *Mycobacterium tuberculosis* . Cell Mol Immunol. 2020;17(9):901‐913. 32728204 10.1038/s41423-020-0502-z PMC7608469

[33] Chandra P , Grigsby SJ , Philips JA. Immune evasion and provocation by *Mycobacterium tuberculosis* . Nat Rev Microbiol. 2022;20(12):750‐766. 35879556 10.1038/s41579-022-00763-4 PMC9310001

[34] Foo CX , Bartlett S , Chew KY , et al. GPR183 antagonism reduces macrophage infiltration in influenza and SARS‐CoV‐2 infection. Eur Respir J. 2023;61(3):2201306 36396144 10.1183/13993003.01306-2022 PMC9686317

[35] Ngo MD , Bartlett S , Bielefeldt‐Ohmann H , et al. A blunted GPR183/oxysterol axis during dysglycemia results in delayed recruitment of macrophages to the lung during *Mycobacterium tuberculosis* Infection. J Infect Dis. 2022;225(12):2219‐2228. 35303091 10.1093/infdis/jiac102 PMC9200159

[36] Sinha R , Ngo MD , Bartlett S , et al. Pre‐diabetes increases tuberculosis disease severity, while high body fat without impaired glucose tolerance is protective. Front Cell Infect Microbiol. 2021;11:691823. 34295838 10.3389/fcimb.2021.691823 PMC8291147

[37] Gatto D , Paus D , Basten A , Mackay CR , Brink R. Guidance of B cells by the orphan G protein‐coupled receptor EBI2 shapes humoral immune responses. Immunity. 2009;31(2):259‐269. 19615922 10.1016/j.immuni.2009.06.016

[38] Pereira JP , Kelly LM , Xu Y , Cyster JG. EBI2 mediates B cell segregation between the outer and centre follicle. Nature. 2009;460(7259):1122‐1126. 19597478 10.1038/nature08226 PMC2809436

[39] Baptista AP , Gola A , Huang Y , et al. The chemoattractant receptor Ebi2 drives intranodal naive CD4(+) T cell peripheralization to promote effective adaptive immunity. Immunity. 2019;50(5):1188‐1201.e6. 31053504 10.1016/j.immuni.2019.04.001

[40] Gatto D , Wood K , Caminschi I , et al. The chemotactic receptor EBI2 regulates the homeostasis, localization and immunological function of splenic dendritic cells. Nat Immunol. 2013;14(5):446‐453. 23502855 10.1038/ni.2555

[41] Yi T , Cyster JG. EBI2‐mediated bridging channel positioning supports splenic dendritic cell homeostasis and particulate antigen capture. Elife. 2013;2:e00757. 23682316 10.7554/eLife.00757 PMC3654440

[42] Shen ZJ , Hu J , Kashi VP , et al. Epstein‒Barr virus‐induced gene 2 mediates allergen‐induced leukocyte migration into airways. Am J Respir Crit Care Med. 2017;195(12):1576‐1585. 28125291 10.1164/rccm.201608-1580OC PMC5476910

[43] Bohrer AC , Castro E , Tocheny CE , et al. Rapid GPR183‐mediated recruitment of eosinophils to the lung after *Mycobacterium tuberculosis* infection. Cell Rep. 2022;40(4):111144. 35905725 10.1016/j.celrep.2022.111144 PMC9460869

[44] Chu C , Moriyama S , Li Z , et al. Anti‐microbial functions of group 3 innate lymphoid cells in gut‐associated lymphoid tissues are regulated by G‐protein‐coupled receptor 183. Cell Rep. 2018;23(13):3750‐3758. 29949760 10.1016/j.celrep.2018.05.099 PMC6209103

[45] Ardain A , Domingo‐Gonzalez R , Das S , et al. Group 3 innate lymphoid cells mediate early protective immunity against tuberculosis. Nature. 2019;570(7762):528‐532. 31168092 10.1038/s41586-019-1276-2 PMC6626542

[46] Nevius E , Pinho F , Dhodapkar M , et al. Oxysterols and EBI2 promote osteoclast precursor migration to bone surfaces and regulate bone mass homeostasis. J Exp Med. 2015;212(11):1931‐1946. 26438360 10.1084/jem.20150088 PMC4612084

[47] Frascoli M , Ferraj E , Miu B , et al. Skin γδ T cell inflammatory responses are hardwired in the thymus by oxysterol sensing via GPR183 and calibrated by dietary cholesterol. Immunity. 2023;56(3):562‐575.e6. 36842431 10.1016/j.immuni.2023.01.025 PMC10306310

[48] Ulrich AK , Moua NM , Mack A , et al. Meeting report on an integrated research agenda for mosquito‐borne arboviruses. Open Forum Infect Dis. 2025;12(7):ofaf395. 40708628 10.1093/ofid/ofaf395 PMC12287632

[49] Kleynhans L , Kunsevi‐Kilola C , Tshivhula H , et al. Human alveolar macrophage function is impaired in tuberculosis contacts with diabetes. EBioMedicine. 2025;122:106050. 41330113 10.1016/j.ebiom.2025.106050 PMC12719684

[50] Saula AY , Cevik M , Cliff JM , Ronacher K , Bowness R. Immune dysregulation in tuberculosis‒diabetes comorbidity: mechanistic and translational insights. Front Immunol. 2026;17:1803046. 42112381 10.3389/fimmu.2026.1803046 PMC13149154

[51] Chalmin F , Rochemont V , Lippens C , et al. Oxysterols regulate encephalitogenic CD4(+) T cell trafficking during central nervous system autoimmunity. J Autoimmun. 2015;56:45‐55. 25456971 10.1016/j.jaut.2014.10.001

[52] Wanke F , Moos S , Croxford AL , et al. EBI2 is highly expressed in multiple sclerosis lesions and promotes early CNS migration of encephalitogenic CD4 T cells. Cell Rep. 2017;18(5):1270‐1284. 28147280 10.1016/j.celrep.2017.01.020

[53] Zhao M , Mei Y , Zhao Z , et al. Abnormal lower expression of GPR183 in peripheral blood T and B cell subsets of systemic lupus erythematosus patients. Autoimmunity. 2022;55(7):429‐442. 35875859 10.1080/08916934.2022.2103119

[54] Taneera J , Mohammed I , Mohammed AK , et al. Orphan G‐protein coupled receptor 183 (GPR183) potentiates insulin secretion and prevents glucotoxicity‐induced β‐cell dysfunction. Mol Cell Endocrinol. 2020;499:110592. 31550518 10.1016/j.mce.2019.110592

[55] Emgård J , Kammoun H , García‐Cassani B , et al. Oxysterol sensing through the receptor GPR183 promotes the lymphoid‐tissue‐inducing function of innate lymphoid cells and colonic inflammation. Immunity. 2018;48(1):120‐132.e8. 29343433 10.1016/j.immuni.2017.11.020 PMC5772175

[56] Wyss A , Raselli T , Perkins N , et al. The EBI2‐oxysterol axis promotes the development of intestinal lymphoid structures and colitis. Mucosal Immunol. 2019;12(3):733‐745. 30742043 10.1038/s41385-019-0140-x

[57] Niss Arfelt K , Barington L , Benned‐Jensen T , et al. EBI2 overexpression in mice leads to B1 B‐cell expansion and chronic lymphocytic leukemia‐like B‐cell malignancies. Blood. 2017;129(7):866‐878. 28003273 10.1182/blood-2016-02-697185 PMC5314812

[58] Weiss G , Schaible UE. Macrophage defense mechanisms against intracellular bacteria. Immunol Rev. 2015;264(1):182‐203. 25703560 10.1111/imr.12266 PMC4368383

[59] Manzanillo PS , Shiloh MU , Portnoy DA , Cox JS. *Mycobacterium tuberculosis* activates the DNA‐dependent cytosolic surveillance pathway within macrophages. Cell Host Microbe. 2012;11(5):469‐480. 22607800 10.1016/j.chom.2012.03.007 PMC3662372

[60] Zumla A , Nahid P , Cole ST . Advances in the development of new tuberculosis drugs and treatment regimens. Nat Rev Drug Discov. 2013;12(5):388‐404. 23629506 10.1038/nrd4001

[61] Lee HJ , Kang SJ , Woo Y , Hahn TW , Ko HJ , Jung YJ. TLR7 stimulation with imiquimod induces selective autophagy and controls *Mycobacterium tuberculosis* growth in mouse macrophages. Front Microbiol. 2020;11:1684. 32765474 10.3389/fmicb.2020.01684 PMC7380068

[62] Watson RO , Bell SL , MacDuff DA , et al. The cytosolic sensor cGAS detects *Mycobacterium tuberculosis* DNA to induce type I interferons and activate autophagy. Cell Host Microbe. 2015;17(6):811‐819. 26048136 10.1016/j.chom.2015.05.004 PMC4466081

[63] Watson RO , Manzanillo PS , Cox JS. Extracellular M. tuberculosis DNA targets bacteria for autophagy by activating the host DNA‐sensing pathway. Cell. 2012;150(4):803‐815. 22901810 10.1016/j.cell.2012.06.040 PMC3708656

[64] Manzanillo PS , Ayres JS , Watson RO , et al. The ubiquitin ligase parkin mediates resistance to intracellular pathogens. Nature. 2013;501(7468):512‐516. 24005326 10.1038/nature12566 PMC3886920

[65] Gluschko A , Farid A , Herb M , Grumme D , Krönke M , Schramm M. Macrophages target Listeria monocytogenes by two discrete non‐canonical autophagy pathways. Autophagy. 2022;18(5):1090‐1107. 34482812 10.1080/15548627.2021.1969765 PMC9196813

[66] Köster S , Upadhyay S , Chandra P , et al. *Mycobacterium tuberculosis* is protected from NADPH oxidase and LC3‐associated phagocytosis by the LCP protein CpsA. Proc Natl Acad Sci U S A. 2017;114(41):E8711‐E8720. 28973896 10.1073/pnas.1707792114 PMC5642705

[67] Wassermann R , Gulen MF , Sala C , et al. *Mycobacterium tuberculosis* differentially activates cGAS‐ and inflammasome‐dependent intracellular immune responses through ESX‐1. Cell Host Microbe. 2015;17(6):799‐810. 26048138 10.1016/j.chom.2015.05.003

[68] Romagnoli A , Etna MP , Giacomini E , et al. ESX‐1 dependent impairment of autophagic flux by *Mycobacterium tuberculosis* in human dendritic cells. Autophagy. 2012;8(9):1357‐1370. 22885411 10.4161/auto.20881 PMC3442882

[69] Kim JS , Lim H , Seo JY , et al. GPR183 regulates 7α,25‐dihydroxycholesterol‐induced oxiapoptophagy in L929 mouse fibroblast cell. Molecules. 2022;27(15):4798 35956750 10.3390/molecules27154798 PMC9369580

[70] Ponnusamy N , Arumugam M . Interaction of host pattern recognition receptors (PRRs) with *Mycobacterium tuberculosis* and ayurvedic management of tuberculosis: a systemic approach. Infect Disord Drug Targets. 2022;22(2):e130921196420. 34517809 10.2174/1871526521666210913110834

[71] Collins AC , Cai H , Li T , et al. Cyclic GMP‐AMP synthase is an innate immune DNA sensor for *Mycobacterium tuberculosis* . Cell Host Microbe. 2015;17(6):820‐828. 26048137 10.1016/j.chom.2015.05.005 PMC4499468

[72] Zeng R , Fang M , Shen A , et al. Discovery of a highly potent oxysterol receptor GPR183 antagonist bearing the benzo[d]thiazole structural motif for the treatment of inflammatory bowel disease (IBD). J Med Chem. 2024;67(5):3520‐3541. 38417036 10.1021/acs.jmedchem.3c01905

[73] Xi J , Gong H , Li Z , et al. Discovery of a first‐in‐class GPR183 antagonist for the potential treatment of rheumatoid arthritis. J Med Chem. 2023;66(23):15926‐15943. 38047891 10.1021/acs.jmedchem.3c01364

