# Supplementary material for: Targeting the GPR183/EBI2‒oxysterol axis in tuberculosis: Immunometabolic regulation and prospects for host‐directed therapy
Source: Clin Transl Med. 2026 Sep 11;16(9):e70755. doi: 10.1002/ctm2.70755 (PMC13570198; doi:10.1002/ctm2.70755)
Supplement: Supplementary file 1 [file CTM2-16-e70755-s001.docx]

**Supplementary Table 1** In the Past Decade: Targeted Class A GPCRs Drugs Approved

| Drugs | Efficacy | Targets | Target Type | Year | Drugs | Efficacy | Targets | Target Type | Year |
| --- | --- | --- | --- | --- | --- | --- | --- | --- | --- |
| ALVERINE | Antispasmodics | 5HT1A | A (Rhodopsin) | 2014 | OXYCODONE | Analgesics | OPRD/K/M | A (Rhodopsin) | 2016 |
| NALOXEGOL | Opioid-Induced Constipation | OPRM | A (Rhodopsin) | 2014 | NALTREXONE | Addiction | OPRD/K/M | A (Rhodopsin) | 2016 |
| NETUPITANT | Antiemetics | NK1R | A (Rhodopsin) | 2014 | PIMAVANSERIN | Antiparkinson | 5HT2A | A (Rhodopsin) | 2016 |
| OLODATEROL | Chronic Obstructive Pulmonary Disease | ADRB2 | A (Rhodopsin) | 2014 | RUPATADINE | Anti-Allergic | HRH1  FTAFR | A (Rhodopsin) | 2016 |
| DROXIDOPA | Orthostatic Hypotension | ADA1A/B/D  ADA2A/2B/2C  ADRB1/2/3 | A (Rhodopsin) | 2014 | FLUTICASONE PROPIONATE AND SALMETEROL | Asthma | β2-AR | A (Rhodopsin) | 2017 |
| SUVOREXANT | Hypnotics And Sedatives | OX1R/2R | A (Rhodopsin) | 2014 | NALDEMEDINE | Opioid-Induced Constipation | OPRM | A (Rhodopsin) | 2017 |
| TASIMELTEON | Hypnotics And Sedatives | MTR1A/1B | A (Rhodopsin) | 2014 | ANGIOTENSIN II | Septic Or Other Distributive Shock | AGTR1 | A (Rhodopsin) | 2017 |
| VORAPAXAR | Cardiovascular Disease | PAR1 | A (Rhodopsin) | 2014 | MACIMORELIN | Adult Growth Hormone Deficiency | GHSR | A (Rhodopsin) | 2017 |
| ARIPIPRAZOLE | Psychosis | 5HT1A/2A  DRD2 | A (Rhodopsin) | 2015 | LATANOPROSTENE BUNOD | Glaucoma Or Ocular Hypertension | PTGFR | A (Rhodopsin) | 2017 |
| LEVODOPA | Antiparkinson | DRD1/2/3/4/5 | A (Rhodopsin) | 2015 | OXYMETAZOLINE HYDROCHLORIDE | Rosacea | α1-AR | A (Rhodopsin) | 2017 |
| ASFOTASE ALFA | Hypophosphatasia | S1PR1 | A (Rhodopsin) | 2015 | CETIRIZINE HYDROCHLORIDE | Conjunctivitis, Allergic | H1R | A (Rhodopsin) | 2017 |
| BREXPIPRAZOLE | Anti-Depressant | 5HT1A/2A/7R  DRD2 | A (Rhodopsin) | 2015 | EPINEPHRINE | Allergic Reactions | α/β-AR | A (Rhodopsin) | 2017 |
| CANGRELOR | Antithrombotic | P2Y12 | A (Rhodopsin) | 2015 | TRIPTORELIN | Precocious Puberty | GnRH | A (Rhodopsin) | 2017 |
| CARIPRAZINE | Schizophrenia | DRD2/3 | A (Rhodopsin) | 2015 | BUPRENORPHINE | Opioid Use Disorder | μ-opioid receptor | A (Rhodopsin) | 2017 |
| ELUXADOLINE | Irritable Bowel Syndrome | OPRD/M | A (Rhodopsin) | 2015 | BRIMONIDINE TARTRATE | Ocular Redness | α2-AR | A (Rhodopsin) | 2017 |
| FLIBANSERIN | Sexual Dysfunction | 5HT1A/2A | A (Rhodopsin) | 2015 | GILTERITINIB | Relapsed Or Refractory Acute Myeloid Leukemia | Serotonin Receptors | A (Rhodopsin) | 2018 |
| ROLAPITANT | Antiemetics | NK1R | A (Rhodopsin) | 2015 | REVEFENACIN | Chronic Obstructive Pulmonary Disease | CHRM1/2/3/4/5 | A (Rhodopsin) | 2018 |
| SELEXIPAG | Antihypertensive | PI2R | A (Rhodopsin) | 2015 | LOFEXIDINE HYDROCHLORIDE | Opiate Withdrawal | α2-AR | A (Rhodopsin) | 2018 |
| BUPRENORPHINE | Anti-Depressant; Analgesics; Opioid Addiction | OPRM/K | A (Rhodopsin) | 2016 | LUTETIUM 177 DOTATATE | Gastroenteropancreatic Neuroendocrine Tumors | SSTR2 | A (Rhodopsin) | 2018 |
| FORMOTEROL | Chronic Obstructive Pulmonary Disease | ADRB2 | A (Rhodopsin) | 2016 | TOLVAPTAN | Adults at risk for autosomal dominant polycystic kidney disease | V2R | A (Rhodopsin) | 2018 |

| Drugs | Efficacy | Targets | Target Type | Year | Drugs | Efficacy | Targets | Target Type | Year |
| --- | --- | --- | --- | --- | --- | --- | --- | --- | --- |
| FOSNETUPITANT/PALONOSETRON | Chemotherapy-Associated Nausea And Vomiting Prevention | NK1R/5-HT3R | A (Rhodopsin) | 2018 | CETIRIZINE HYDROCHLORIDE | Urticaria | H1R | A (Rhodopsin) | 2019 |
| PRUCALOPRIDE | Chronic Idiopathic Constipation | 5-HT4R | A (Rhodopsin) | 2018 | GALLIUM 68 DOTATOC | Diagnostic Agent For Neuroendocrine Tumors | SSTR2 | A (Rhodopsin) | 2019 |
| CANNABIDIOL | Spasticity Related To Multiple Sclerosis And Epilepsy | CNR1 | A (Rhodopsin) | 2018 | ISTRADEFYLLINE | Parkinson’S Disease | ADORA2A | A (Rhodopsin) | 2019 |
| ALBUTEROL SULFATE | Asthma And Copd | β2-AR | A (Rhodopsin) | 2018 | SIPONIMOD | Relapsing Forms Of Multiple Sclerosis | S1PR1/5 | A (Rhodopsin) | 2019 |
| FOSTAMATINIB | Thrombocytopenia Chronic Immune | Multiple Targets, including ADORA3 | A (Rhodopsin) | 2018 | OZANIMOD | Relapsing Forms Of Multiple Sclerosis | S1PR1/5 | A (Rhodopsin) | 2020 |
| MOGAMULIZUMAB-KPKC | Refractory Mycosis Fungoides, Or Sézary Syndrome | CCR4 | A (Rhodopsin) | 2018 | OLICERIDINE | Moderate-To-Severe Acute Pain | OPRM1 | A (Rhodopsin) | 2020 |
| ELAGOLIX | Endometriosis-Associated Moderate-To-Severe Pain | GNRHR | A (Rhodopsin) | 2018 | SETMELANOTIDE | Weight Loss (Obesity/Overweight) | mACh4R | A (Rhodopsin) | 2020 |
| MOGAMULIZUMAB-KPKC | Non-Hodgkin Lymphoma | CXCR4 | A (Rhodopsin) | 2018 | AMISULPRIDE | Nausea/Vomiting, Postoperative | D2R | A (Rhodopsin) | 2020 |
| SUMATRIPTAN | Migraine | 5-HT1B/1D | A (Rhodopsin) | 2019 | RELUGOLIX | Prostate Cancer | GnRH | A (Rhodopsin) | 2020 |
| ACLIDINIUM BROMIDE AND FORMOTEROL FUMARATE | Chronic Obstructive Pulmonary Disease | mAChR/β2-AR | A (Rhodopsin) | 2019 | TRAMADOL HYDROCHLORIDE | Pain | μ-opioid | A (Rhodopsin) | 2020 |
| LASMIDITAN | Migraine | HTR1F | A (Rhodopsin) | 2019 | CLASCOTERONE | Acne | mAChR | A (Rhodopsin) | 2020 |
| PITOLISANT | Narcolepsy, Excessive Daytime Sleepiness | HRH3 | A (Rhodopsin) | 2019 | OZANIMOD | Multiple Sclerosis, Ulcerative Colitis | S1P | A (Rhodopsin) | 2020 |
| AFAMELANOTIDE | Prevention Of Phototoxicity In Erythropoietic Protoporphyria | MC1R | A (Rhodopsin) | 2019 | CYSTEAMINE | Radiation Sickness | NPY2R | A (Rhodopsin) | 2020 |
| LEMBOREXANT | Insomnia | HCRTR1 | A (Rhodopsin) | 2019 | PONESIMOD | Multiple Sclerosis | S1P | A (Rhodopsin) | 2021 |
| LUMATEPERONE | Schizophrenia | HTR2A  DRD1/2 | A (Rhodopsin) | 2019 | VARENICLINE SOLUTION | Dry Eye Disease | mAChR | A (Rhodopsin) | 2021 |
| BREMELANOTIDE | Sexual Arousal Disorder | MC1R | A (Rhodopsin) | 2019 | PILOCARPINE OPHTHALMIC | Presbyopia | mAChR | A (Rhodopsin) | 2021 |
| PHENYLEPHRINE HYDROCHLORIDE | Hypotension | α1-AR | A (Rhodopsin) | 2019 | DIFELIKEFALIN | Pain Following Abdominal Surgery | κ-opioid receptor | A (Rhodopsin) | 2021 |

| Drugs | Efficacy | Targets | Target Type | Year | Drugs | Efficacy | Targets | Target Type | Year |
| --- | --- | --- | --- | --- | --- | --- | --- | --- | --- |
| AVACOPAN | Severe Active Anti-Neutrophil Cytoplasmic Autoantibody-Associated Vasculitis | C5aR | A (Rhodopsin) | 2021 | PHENYLEPHRINE HYDROCHLORIDE AND TROPICAMIDE | Pupillary Dilation | α1-AR | A (Rhodopsin) | 2023 |
| NALOXONE HYDROCHLORIDE | Opioid Emergency | [Opioid receptor](https://www.medchemexpress.cn/Targets/Opioid%20Receptor.html) | A (Rhodopsin) | 2021 | PHENTOLAMINE MESYLATE | Mydriasis | α-AR | A (Rhodopsin) | 2023 |
| RELUGOLIX | Premenopausal Women To Control Heavy Menstrual Bleeding Due To Uterine Fibroids | GnRH | A (Rhodopsin) | 2021 | HYDROMORPHONE HYDROCHLORIDE | Analgesics | OPRM  Opioid receptor | A (Rhodopsin) | 2023 |
| LEUPROLIDE | Adult Patients With Advanced Prostate Cancer | GnRH | A (Rhodopsin) | 2021 | ETRASIMOD ARGININE | Ulcerative Colitis | S1PR1 | A (Rhodopsin) | 2023 |
| DARIDOREXANT | Insomnia | OXR | A (Rhodopsin) | 2022 | VASOPRESSIN | Hypotension | V1aR/3R/2R | A (Rhodopsin) | 2023 |
| DEXMEDETOMIDINE | Agitation | α2-AR | A (Rhodopsin) | 2022 | TRAVOPROST | Ocular Hypertension、Open-Angle Glaucoma | PF2R  FP | A (Rhodopsin) | 2023 |
| TERLIPRESSIN | Hepatorenal Syndrome | V1R/2R | A (Rhodopsin) | 2022 | ILOPROST | Frostbite | PGI2R | A (Rhodopsin) | 2024 |
| OMIDENEPAG ISOPROPYL | Glaucoma And Ocular Hypertension | EP2 | A (Rhodopsin) | 2022 | APROCITENTAN | Hypertension | ETA/ETB | A (Rhodopsin) | 2024 |
| NALOXONE | The Reversal Of An Opioid Overdose | μ-opioid receptor | A (Rhodopsin) | 2023 | MACITENTAN | Pulmonaryarterial Hypertension | ETA/ETB | A (Rhodopsin) | 2024 |
| SPARSENTAN | Primary Immunoglobulin A Nephropathy | AT1R/ETAR | A (Rhodopsin) | 2023 | RISPERIDONE | Schizophrenia | DRD2  5-HT2A | A (Rhodopsin) | 2024 |
| ALBUTEROL AND BUDESONIDE | Asthma | β2-AR | A (Rhodopsin) | 2023 | VALSARTAN | Chronic Heart Faiture | Angiotensin II Receptor | A (Rhodopsin) | 2024 |
| RIZATRIPTAN | Migraine | 5-HT1B/1DR | A (Rhodopsin) | 2023 | NALOXONE HYDROCHLORIDE | Reversal Of The Effects Of Opioid Drugs | [Opioid receptor](https://www.medchemexpress.cn/Targets/Opioid%20Receptor.html) | A (Rhodopsin) | 2024 |
| PILOCARPINE HYDROCHLORIDE | Presbyopia | M3R | A (Rhodopsin) | 2023 | BRIMONIDINE TARTRATE | Ocular Redness | α2-AR | A (Rhodopsin) | 2024 |
| FEZOLINETANT | Menopausal Disorders, Hot Flashes | NK3R | A (Rhodopsin) | 2023 | DIAZEPAM | Epilepsy | Dopamine receptor | A (Rhodopsin) | 2024 |
| NALMEFENE HYDROCHLORIDE | Opioid Overdose | μ-opioid receptor | A (Rhodopsin) | 2023 | MAVORIXAFOR | Whim Syndrome | CXCR4 | A (Rhodopsin) | 2024 |
| MOTIXAFORTIDE ACETATE | Multiple Myeloma | CXCR4 | A (Rhodopsin) | 2023 | CLONIDINE HYDROCHLORIDE | Attention-Deficit/Hyperactivity-Disorder | α2-AR | A (Rhodopsin) | 2024 |
| GEPIRONE HYDROCHLORIDE | Anti-Depressant | 5-HT1A/2A | A (Rhodopsin) | 2023 | VORTIOXETINE | Anti-Depressant | 5-HT1A/1B/3A/7 | A (Rhodopsin) | 2024 |

**Supplementary Table 2** In the Past Decade: Targeted Class B1 GPCRs Drugs Approved

| Drugs | Efficacy | Targets | Target Type | Year | Drugs | Efficacy | Targets | Target Type | Year |
| --- | --- | --- | --- | --- | --- | --- | --- | --- | --- |
| ALBIGLUTIDE、  DULAGLUTIDE | Antidiabetic | GLP1R | B1 (Secretin) | 2014 | SEMAGLUTIDE (ORAL) | Type 2 Diabetes Mellitus | GLP-1R | B1 (Secretin) | 2019 |
| DULAGLUTIDE | Antidiabetic | GLP1R | B1 (Secretin) | 2014 | UBROGEPANT | Migraine | CGRP | B1 (Secretin) | 2019 |
| PARATHYROID HORMONE | Anti-Osteoporotic | PTH1R  PTH2R | B1 (Secretin) | 2015 | RIMEGEPANT | Migraine | CALCRL | B1 (Secretin) | 2020 |
| PARATHYROID HORMONE | Hypoparathyroidism  Control Hypocalcemia | PTH1R | B1 (Secretin) | 2015 | EPTINEZUMAB-JJMR | Migraine (Prevention) | CALCRL | B1 (Secretin) | 2020 |
| LIXISENATIDE | Antidiabetic | GLP1R | B1 (Secretin) | 2016 | ATOGEPANT | Prevent Migraine Headache | CGRP | B1 (Secretin) | 2021 |
| ABALOPARATIDE | Anti-Osteoporotic | PTH1R | B1 (Secretin) | 2017 | TIRZEPATIDE | Type 2 Diabetes In Adults To Decrease Blood Sugar Levels | GLP-1 | B1 (Secretin) | 2022 |
| EXENATIDE | Type 2 Diabetes | GLP-1 | B1 (Secretin) | 2017 | ZAVEGEPANT | Migraine | CGRP | B1 (Secretin) | 2023 |
| SEMAGLUTIDE (INJECTION) | Type 2 Diabetes, Cardiovascular Risk Reduction | GLP-1R | B1 (Secretin) | 2017 | TIRZEPATIDE | Anti-Obesity、Antidiabetic | GLP-1R | B1 (Secretin) | 2023 |
| GALCANEZUMAB-GNLM | Migraine Prevention, Cluster Headaches | CGRP | B1 (Secretin) | 2018 | TERIPARATIDE | Osteoporosis | PTH1R | B1 (Secretin) | 2024 |
| ERENUMAB-AOOE | Migraine (Prevention) | CALCRL | B1 (Secretin) | 2018 |  |  |  |  |  |

**Supplementary Table 3** In the Past Decade: Targeted Class C GPCRs Drugs Approved

| Drugs | Efficacy | Targets | Target Type | Year | Drugs | Efficacy | Targets | Target Type | Year |
| --- | --- | --- | --- | --- | --- | --- | --- | --- | --- |
| ETELCALCETIDE | Parathyroid Disorders | CASR | C (Glutamate) | 2017 | GANAXOLONE | Cdkl5 Deficiency Disorder | GABA | C (Glutamate) | 2022 |
| BREXANOLONE | Postpartum Depression | GABA | C (Glutamate) | 2019 | ZURANOLONE | Postpartum Depression | GABAA | C (Glutamate) | 2023 |
| BACLOFEN | Spasticity | GABBR1 | C (GABA) | 2021 |  |  |  |  |  |

**Supplementary Table 4** In the Past Decade: Targeted Class F GPCRs Drugs Approved

| Drugs | Efficacy | Targets | Target Type | Year | Drugs | Efficacy | Targets | Target Type | Year |
| --- | --- | --- | --- | --- | --- | --- | --- | --- | --- |
| SONIDEGIB | Antineoplastic | SMO | F (Frizzled) | 2015 | GLASDEGIB | Acute Myeloid Leukemia | SMO | F (Frizzled) | 2018 |

Note: This table includes drugs identified and compiled manually from the original NDA and BLA databases at Drugs@FDA, as well as those obtained from the GPCRdb database and published literature.
